# Prevalence of disease-causing genes in Japanese patients with *BRCA1/2*-wildtype hereditary breast and ovarian cancer syndrome

**DOI:** 10.1038/s41523-020-0163-1

**Published:** 2020-06-12

**Authors:** Tomoko Kaneyasu, Seiichi Mori, Hideko Yamauchi, Shozo Ohsumi, Shinji Ohno, Daisuke Aoki, Shinichi Baba, Junko Kawano, Yoshio Miki, Naomichi Matsumoto, Masao Nagasaki, Reiko Yoshida, Sadako Akashi-Tanaka, Takuji Iwase, Dai Kitagawa, Kenta Masuda, Akira Hirasawa, Masami Arai, Junko Takei, Yoshimi Ide, Osamu Gotoh, Noriko Yaguchi, Mitsuyo Nishi, Keika Kaneko, Yumi Matsuyama, Megumi Okawa, Misato Suzuki, Aya Nezu, Shiro Yokoyama, Sayuri Amino, Mayuko Inuzuka, Tetsuo Noda, Seigo Nakamura

**Affiliations:** 1 Project for Development of Innovative Research on Cancer Therapeutics, Cancer Precision Medicine Center, Japanese Foundation for Cancer Research, 3-8-31 Ariake, Koto-ku Tokyo, Japan; 2 Department of Breast Surgical Oncology, St. Luke’s International Hospital, 10-1 Akashi-cho, Chuo-ku Tokyo, Japan; 3 National Hospital Organization Shikoku Cancer Center, 160 Kou, Minamiumemoto-machi, Matsuyama, Ehime Japan; 4 Breast Oncology Center, Cancer Institute Hospital, Japanese Foundation for Cancer Research, 3-8-31 Ariake, Koto-ku Tokyo, Japan; 5 Department of Obstetrics & Gynecology, Keio University School of Medicine, 35 Shinano-cho, Shinjuku-ku Tokyo, Japan; 6 Sagara Hospital, 3-31 Matsubara-cho, Kagoshima, Japan; 7 Department of Human Genetics, Yokohama City University Graduate School of Medicine, Fukuura 3-9, Kanazawa-ku Yokohama, Japan; 8 Department of Integrative Genomics, Tohoku Medical Megabank Organization, Tohoku University, 2-1, Seiryo-machi, Aoba-ku, Sendai, Miyagi Japan; 9 Department of Clinical Genetic Oncology, Cancer Institute Hospital, Japanese Foundation for Cancer Research, 3-8-31 Ariake, Koto-ku Tokyo, Japan; 10 Division of Breast Surgical Oncology, Showa University School of Medicine, 1-5-8 Hatanodai, Shinagawa-ku Tokyo, Japan; 11 Cancer Institute, Japanese Foundation for Cancer Research, 3-8-31 Ariake, Koto-ku Tokyo, Japan

**Keywords:** Surgical oncology, Breast cancer

## Abstract

Panel sequencing of susceptibility genes for hereditary breast and ovarian cancer (HBOC) syndrome has uncovered numerous germline variants; however, their pathogenic relevance and ethnic diversity remain unclear. Here, we examined the prevalence of germline variants among 568 Japanese patients with *BRCA1/2*-wildtype HBOC syndrome and a strong family history. Pathogenic or likely pathogenic variants were identified on 12 causal genes for 37 cases (6.5%), with recurrence for 4 SNVs/indels and 1 CNV. Comparisons with non-cancer east-Asian populations and European familial breast cancer cohorts revealed significant enrichment of *PALB2*, *BARD1*, and *BLM* mutations. Younger onset was associated with but not predictive of these mutations. Significant somatic loss-of-function alterations were confirmed on the wildtype alleles of genes with germline mutations, including *PALB2* additional somatic truncations. This study highlights Japanese-associated germline mutations among patients with *BRCA1/2* wildtype HBOC syndrome and a strong family history, and provides evidence for the medical care of this high-risk population.

## Introduction

Germline deleterious mutations in *BRCA1* or *BRCA2* alleles are associated with a considerably increased risk of developing breast and/or ovarian cancer. This knowledge has prompted preventive medical care regimes, such as surveillance and risk-reducing surgery, for those who carry these mutations. A mutation not only in *BRCA1*/*2* but also in several other genes, including *TP53*, *PTEN*, and *CDH1* (with high penetrance), and *ATM*, *CHEK2*, and *PALB2* (with moderate penetrance), can lead to hereditary breast and ovarian cancer (HBOC) syndrome, which has been linked with different levels of risk and prevalence in the population^1,2^. Recent advances in sequencing technology have facilitated multigene panel genetic testing and led to the identification of numerous variants of disease-causing genes. However, the risks associated with most of these variants are largely unknown, and it is therefore difficult to make decisions in clinical practice as to whether there is a need for further medical care. Moreover, the ethnicity-related differences in the prevalence of variants^3^ add an additional layer of difficulty in evaluating the pathogenicity of rare variants, particularly among non-Caucasian populations, which are less-well studied.

Several mutations, including those in *BRCA1*, *BRCA2*, *ATM*, *CHEK2*, and *PALB2*, have been associated with the clinicopathological features exemplified by the breast cancer subtype and tissue of origin for additional cancers^1,4^. Although there is rarely perfect agreement in such genotype–phenotype links, the link remains important, as phenotype presentations can support the validity of undertaking genetic analyses and subsequent pathogenicity interpretations^5^. By the same context, loss of heterozygosity (LOH) in the wildtype allele in a tumor through copy loss or additional somatic truncation (AST) may help to implicate the importance of the detected variant in the tumorigenic process^1,6^. In this study, we report the frequencies of germline variants in previously recognized causal genes for HBOC syndrome across 568 Japanese patients with wildtype (i.e., mutation-negative) *BRCA1*/*2* genes and a strong family history. These classifications were associated with clinicopathological data, and were verified by comparing the identified alleles in our cohort against those in germline-variant databases and against genetic alterations in tumor specimens.

## Results

### Study cohort and clinicopathological information

**Fig. 1:**
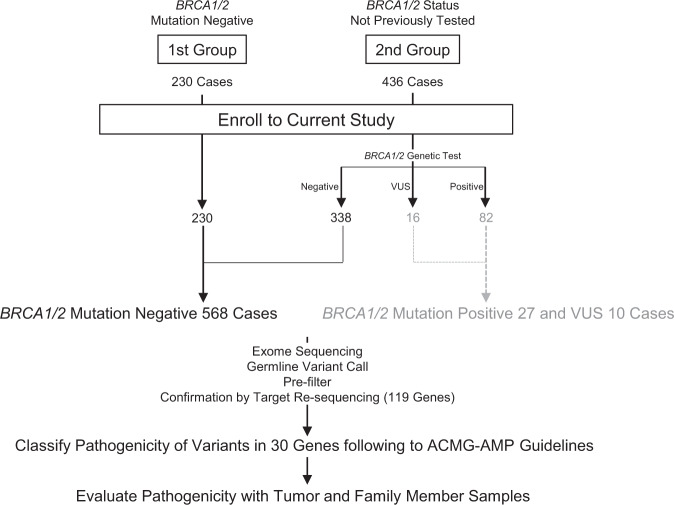
Study design. Samples derived from all *BRCA1/2* WT patients (568 cases) were rendered to exome sequencing and further germline-variant analyses and interpretations. To ascertain consistency between the results derived from our pathogenicity classification and those from commercial *BRCA1/2* genetic tests, we also exome-sequenced *BRCA1/2* mutation-positive samples and variants of uncertain significance (VUS) samples (27 and 10 samples, respectively, in gray font).

**Fig. 2:**
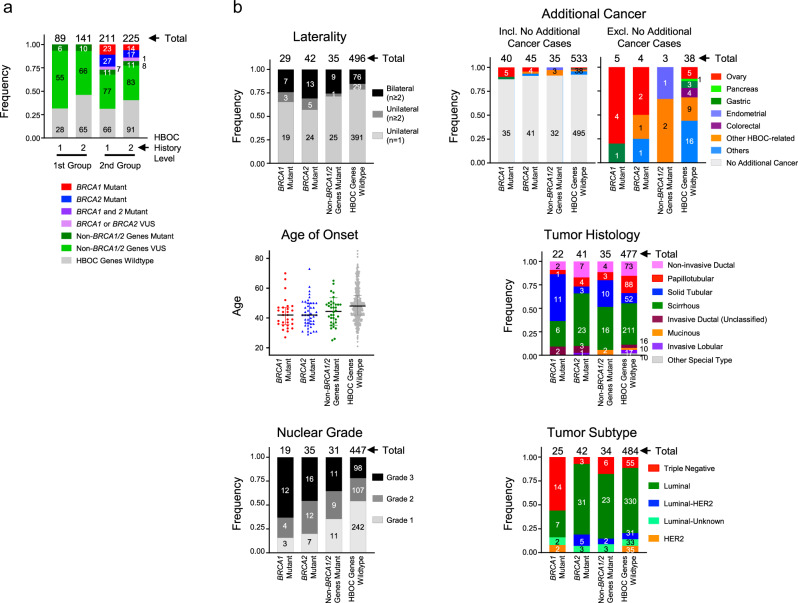
Clinicopathological properties of patients and patient tumors bearing *BRCA1* or *BRCA2* mutations, non-*BRCA1/2* gene mutations or WT genes. **a** Frequencies of *BRCA1* mutations, *BRCA2* mutations, other gene mutations (*BRCA1/2* WT) in the different groups. Two levels of criteria were used to stratify patients based on the frequency of breast and/or ovarian cancer in family members (see Methods and Supplementary Note 1—HBOC history levels 1 and 2). Among the 211 level-1 and 225 level-2 cases who received *BRCA1/2* genetic tests, 50 and 31 patients, respectively, had *BRCA1* and/or *BRCA2* mutations (*BRCA1*; 8.5%, *BRCA2*; 10.1% and both; 0.2% in total 436 patients). One case (HBOC history level-2) had a *BRCA1*/*BRCA2* double mutation. The frequencies of mutations in other genes (*BRCA1/2* WT) are shown. **b** Top left panel: Laterality. Unilateral (*n* = 1), Unilateral (*n* ≥ 2) and Bilateral (*n* ≥ 2) indicate one unilateral occurrence, at least two unilateral occurrences, and at least two bilateral occurrences of primary breast cancer, respectively. The tumors were defined as independent primaries (not local recurrent tumors) when cancer cells were absent in the surgical margin of the first tumor, and also when a difference could be seen in the position of occurrence, histology, hormonal status and HER2 expression of the second tumor, according to previous criteria^49^. Top right panels: Additional cancer. The frequencies of tissue cancer other than breast cancer are shown including (top right-left) and excluding (top right-right) no additional cancer cases. Middle left panel: Age of onset. Middle right panel: Tumor histology. Bottom left panel: Nuclear grade. Bottom right panel: Tumor subtype. For each panel, the number of tumors for each category is shown on and above the bars. Incl. including, Excl. excluding.

Informed consent was obtained from 666 patients. Patients were recruited and underwent genetic counseling at one of six Japanese institutions (see “Methods”; Fig. 1). Patients were classified into two groups: Group 1 patients (*n* = 230) were negative for *BRCA1/2* mutations (hereafter referred to as *BRCA1/2* WT [wildtype]), whereas group 2 patients (*n* = 436) had not yet received *BRCA1/2* genetic tests. Group 2 patients were subjected to *BRCA1/2* mutational analysis (Methods), of which 82 patients were positive for *BRCA1/2* mutations and 16 were identified as having variants of unknown significance (VUS). *BRCA1* and *BRCA2* mutation single-positive and double-positive cases were enriched (37 [8.5%] for *BRCA1*, 44 [10.1%] for *BRCA2*, and 1 [0.2%] for *BRCA1/2*) among group 2, which indicates sufficient stringency of the inclusion criteria (see also Supplementary Note 1 and Supplementary Fig. 1), with similar prevalence to previous work^7^. The clinicopathological properties associated with *BRCA1* or *BRCA2* mutations versus *BRCA1/2* WT were also highly consistent with previous literature (Fig. 2)^4,8–13^.

Group 1 (*n* = 230 cases) and 2 (*n* = 338) WT cases were combined for further analyses. Of the 568 *BRCA1/2* WT cases, 534 (94%) patients had breast carcinomas (532 females and 2 males); 29 (5.1%) had ovarian carcinomas, including peritoneal and fallopian tube carcinomas; and 5 (0.9%) had “synchronous” breast and ovarian carcinomas (both cancers were diagnosed at the same time).

### Variant classifications for 28 disease-causing genes in *BRCA1/2* WT cases

Germline DNA samples for the 568 *BRCA1/2* WT cases were subsequently exome sequenced; exome analyses were also performed for 27 mutation-positive and 10 VUS cases selected at random from the cohort to determine the concordance of our pathogenicity classifications with commercial genetic tests (see Methods and Supplementary Note 2). We confirmed all of the sequenced cases were unrelated. Exome sequencing yielded a median 123-fold coverage, with 90% of bases meeting the >20-fold coverage threshold for variant detection.

Across the 568 *BRCA1/2* WT cases, germline mutation calling with UnifiedGenotyper and HaplotypeCaller^14^ detected 259,742 variants (240,015 single nucleotide variants [SNVs] and 19,727 indels on 28 HBOC susceptibility genes, excluding *BRCA1/2*; Supplementary Methods and Supplementary Table 1)^13^. After filtering, 524 variants (491 SNVs and 33 indels) on 28 genes remained in 345 (60.7%) cases (Supplementary Fig. 2). These variants were subjected to a 5-category pathogenicity classification pipeline according to the American College of Medical Genetics and Genomics–Association for Molecular Pathology (ACMG-AMP) guidelines (Supplementary Note 2 and Supplementary Fig. 3; Methods). This classification resulted in 5 pathogenic (P), 30 likely pathogenic (LP), 446 VUS, 40 likely benign (LB), and 4 benign (B) assignments in 4, 13, 29, 10, and 3 genes, respectively (Supplementary Fig. 4)^15^. In addition, germline copy number variant (CNV) searches using XHMM (ver. 1.0) identified 3 large deletion variants (one *BARD1* exon 5–7 deletion and two *RAD51C* exon 6–9 deletion; Supplementary Fig. 4).

Among the 568 *BRCA1/2* WT cases, 37 (6.5%) cases harbored 38 germline loss-of-function mutations in 12 genes (35 truncations [19 frameshift indels, 13 stop-gain SNVs/indels and 3 splice site SNVs] and 3 large deletions in copy number) (Supplementary Fig. 4 and Table 1)^13,15^. Notably, one case had two P/LP variants on two genes (A0331; *BARD1* p.R150* and *ATM* p.A2626fs; Supplementary Fig. 4 and Table 1)^13,15^. After manual review, all of the variants were assigned to P or LP (Supplementary Fig. 4 and Table 1)^13,15^. Among these 37 cases, 35 had breast cancer and 2 (*ATM* p.R805* and *RAD51D* p.K111fs) had ovarian cancer (Supplementary Fig. 4 and Table 1)^13,15^.

**Table 1:** Frequencies of pathogenic/likely pathogenic variants in the study cohort and in the disease and population databases.

Variant					Disease DB		Population DB		
Case	Gene	Nucleotide substitution	Amino Acid substitution	ClinVar	HGMD	HGVD (Japanese)	TMM (Japanese)	ExAC (East Asian)	ExAC (Other Ethnicity)
A1013	*PALB2*	c.2167_2168del	p.M723fs	Present	Present	NR	NR	NR	7/98344
A0139	*PALB2*	c.C3256T	p.R1086*	Present	Present	NR	NR	NR	1/98344
B0215	*PALB2*	c.C3256T	p.R1086*	Present	Present	NR	NR	NR	1/98344
A0214	*PALB2*	c.G1384T	p.E462*	NR	NR	NR	NR	NR	NR
B0219	*PALB2*	c.G1384T	p.E462*	NR	NR	NR	NR	NR	NR
B0277	*PALB2*	c.T1451G	p.L484*	NR	NR	NR	NR	NR	NR
A0338	*PALB2*	c.820dupA	p.T274fs	NR	NR	NR	NR	NR	NR
D0241	*ATM*	c.C2413T	p.R805*	Present	Present	NR	NR	3/7844	2/98354
E0125	*ATM*	c.240dupA	p.P80fs	NR	NR	NR	NR	NR	NR
A0346	*ATM*	c.1121_1122del	p.Q374fs	NR	NR	2/600	1/7108	NR	NR
B0242	*ATM*	c.4776+2T>A	Splicing	NR	Present	NR	NR	NR	NR
C0158	*ATM*	c.5509_5510del	p.F1837fs	NR	NR	NR	NR	NR	NR
A0301	*BARD1*	c.C1921T	p.R641*	Present	Present	NR	NR	NR	1/98342
A0331	*BARD1*	c.C448T	p.R150*	Present	NR	NR	NR	NR	1/98348
	*ATM*	c.7878_7882del	p.A2626fs	Present	Present	NR	4/7108	NR	NR
A0298	*BARD1*	c.C1345T	p.Q449*	NR	NR	NR	NR	NR	NR
B0258	*BARD1*	c.518dupC	p.A173fs	NR	NR	NR	NR	NR	NR
E0114	*BARD1*	Exon 5–7 Deletion	NR	NR	NA	NA	NR	NR	
C0120	*RAD51D*	c.331_332insTA	p.K111fs	Present	Present	1/858	5/7108	9/7866	NR
D0239	*RAD51D*	c.331_332insTA	p.K111fs	Present	Present	1/858	5/7108	9/7866	NR
A0231	*RAD51D*	c.454delG	p.V152fs	NR	NR	NR	NR	NR	NR
B0220	*RAD51D*	c.C445T	p.Q149*	NR	NR	NR	NR	NR	NR
D0221	*BLM*	c.319dupT	p.S106fs	NR	NR	1/858	1/7108	1/7856	NR
C0256	*BLM*	c.319dupT	p.S106fs	NR	NR	1/858	1/7108	1/7856	NR
B0187	*BLM*	c.1536dupA	p.G512fs	Present	Present	NR	4/7108	NR	13/98522
B0292	*BLM*	c.3751+2T>C	Splicing	NR	NR	NR	1/7102	NR	NR
A0281	*BRIP1*	c.C1066T	p.R356*	Present	Present	NR	NR	NR	NR
B0285	*BRIP1*	c.3240dupT	p.A1081fs	NR	NR	1/858	2/7108	1/7866	NR
B0170	*BRIP1*	c.918+2T>C	Splicing	NR	NR	NR	NR	NR	NR
E0237	*RAD51C*	c.G133T	p.E45*	NR	NR	NR	NR	NR	NR
C0206	*RAD51C*	Exon 6–9 Deletion	NR	NR	NA	NA	NR	NR	
B0263	*RAD51C*	Exon 6–9 Deletion	NR	NR	NA	NA	NR	NR	
B0288	*FANCM*	c.2190_2191insCT	p.Q730fs	NR	NR	NR	NR	NR	NR
D0231	*FANCM*	c.2521_2524del	p.K841fs	NR	NR	1/858	1/7108	NR	2/98370
E0131	*RAD50*	c.1633dupA	p.D544fs	NR	NR	NR	NR	NR	NR
B0198	*NF1*	c.765delT	p.G255fs	NR	NR	NR	NR	NR	NR
A0277	*CHEK2*	c.1455dupG	p.L486fs	NR	NR	NR	NR	NR	NR
D0222	*RECQL*	c.1548dupT	p.D517_S518delins*	NR	NR	NR	1/7108	NR	NR

Presence or absence of registration in a disease database (ClinVar and HGMD) and in a population database (HGVD, TMM and ExAC; the ExAC data was subdivided into “East Asian” and “Other” ethnicities. Presence or frequencies are shown separately). Fractions are used to show the minor allele frequencies; the top and bottom of each fraction indicates the alternative allele and total allele counts, respectively.

*NR* not registered, *NA* not available.

Of the 27 *BRCA1/2* mutation-positive cases, there were 0 P/LP, 9 VUS and 6 LB/B variants on the 28 genes (excluding *BRCA1/2*). For the 10 *BRCA1/2* VUS cases, there were 1 P/LP (*BRIP1* c.C1315T p.R439*), 9 VUS, and 0 LB/B variants. We show that 31 of the 35 truncations occurred once and were distributed throughout the protein-coding region, suggestive of variant heterogeneity in the genes (Supplementary Fig. 5). Many truncations were in front or in the middle of functionally relevant protein domains. Four SNVs/indels (*PALB2*:p.R1986*, *PALB2*:p.E462*, *RAD51D*:p.K111fs and *BLM*:p.S106fs) and one CNV (*RAD51C* Exon 6–9 deletion) were recurrently detected, suggestive of an ancestral relationship (Supplementary Fig. 5; Table 1)^13^.

### Frequencies of variants in disease and population databases

Among the 38 (33 unique) variants, 19 (16 unique) P/LP SNVs/indels (0 CNVs) had been previously registered in at least one of the databases searched (see Methods; The Cancer Genome Atlas [TCGA] data were excluded in the current study). Three (2 unique) CNVs were not registered in ExAC, which included an east-Asian population. Our searches left us with 16 (15 unique) novel SNVs/indels/CNVs: 3 *PALB2*, 2 *ATM*, 2 *BARD1*, 2 *RAD51D*, 1 *BRIP1*, 1 *RAD51C*, 1 *FANCM*, 1 *RAD50*, 1 *NF1*, and 1 *CHEK2* variants^15^. We surmise that these SNVs/indels and CNVs are specific to Japanese/east-Asian HBOC patients (Table 1)^13^. Moreover, 9 (7 unique) variants, including the recurrently detected *RAD51D*:p.K111fs and *BLM*:p.S106fs, were observed only in Japanese/east-Asian cohorts (HGVD, TMM or east-Asian ExAC) (Table 1)^13^. No pathogenic variants were detected for syndromic, high-penetrant breast cancer susceptibility genes (*TP53*, *PTEN*, *STK11*, and *CDH1*).

### Enrichment of *PALB2*, *BARD1*, *BLM*, and *ATM* mutations in Japanese HBOC cases

To determine mutation enrichment in our cohort, we conducted Fisher exact tests of the germline data from our 568 patients against metadata for 8,695 cases compiled from three databases (HGVD, TMM, and east-Asian ExAC). The “other ethnic” ExAC data were excluded to avoid any ethnicity-related bias (Table 2a)^13^. Noteworthy, allele count information for ethnicity combined with gender was not available from these databases; therefore, the comparison includes male data. Fisher analyses were performed at the gene level but not at the variant level due to the small number of detected variants in the current cohort. We used SNVs/indels but not CNVs, since HGVD and TMM lacked CNV allele count data.

**Table 2:** Case-control analyses of mutated genes in the current cohort compared with population data.

Gene	Number of mutant alleles	Odds ratio	*p*-value	95% confidence intervals	
				Lower	Upper
*(a) Odds ratio of mutated genes in the current cohort compared with the East-Asian population data, including male data*^a^					
*PALB2*	7	10.7	<0.01	3.5	31.3
*BARD1*	4	10.2	<0.01	2.1	43.2
*BLM*	4	3.6	0.04	0.9	11.0
*ATM*	6	2.7	0.03	0.9	6.5
*BRIP1*	2	2.4	NS	0.3	10.4
*RAD51D*	4	1.8	NS	0.5	5.2
*RAD51C*	1	1.7	NS	0.0	13.0
*RECQL*	1	1.5	NS	0.0	10.7
*FANCM*	2	1.4	NS	0.2	5.6
*CHEK2*	1	1.3	NS	0.0	8.5
*RAD50*	1	0.3	NS	0.0	1.8
*NF1*	1	0.2	NS	0.0	1.3
*(b) Odds ratio of mutated genes in the current cohort compared with female only ExAC data without a distinction of ethnicity*^b^					
*PALB2*	7	8.9	<0.01	3.3	20.6
*BARD1*	4	14.8	<0.01	3.4	50.0
* BLM*	4	3.4	0.04	0.9	9.3
*ATM*	6	3.6	0.01	1.3	8.2
*BRIP1*	2	2.9	NS	0.3	11.5
*RAD51D*	4	0.6	NS	0.2	1.5
*RAD51C*	1	2.0	NS	0.0	12.7
*RECQL*	1	0.8	NS	0.0	4.5
*FANCM*	2	0.5	NS	0.1	1.9
*CHEK2*	1	0.4	NS	0.0	2.0
*RAD50*	1	0.2	NS	0.0	1.2
*NF1*	1	0.1	NS	0.0	0.7

ExAC data were excluding the Cancer Genome Atlas (TCGA) data. Allele count information for gender was not available for HGVD or TMM. ExAC provides allele count data for gender and ethnicity separately but not together. The use of similar ethnicity data as the control prohibits further filtering of the allele count data based on gender, and vice versa.

*HGMD* Human Gene Mutation Database, *HGVD* Human Genetic Variation Database, *TMM* Tohoku Medical Megabank Project, *ExAC* Exome Aggregation Consortium, *NS* Non-significant.

^a^Data were combined from HGVD (1208 Japanese), TMM (3554 Japanese), and East-Asian ExAC (3933 east Asian people) (total 8695; all databases include male data) databases and used as the control to compute the odds ratios for each gene (not for each variant).

^b^Female ExAC without distinction of ethnicity (22,937 females) was used as the control to compute the odds ratio for each gene (not for each variant). *p*-values and odds ratios (Fisher exact test) for each gene are shown with upper and lower 95% confidence intervals.

Ten of the 12 genes with truncating SNVs/indels showed positive enrichment in the study population (odds ratio; OR > 1), with significant enrichment for *PALB2*, *BARD1*, *BLM*, and *ATM* (Table 2a)^13^. Fisher exact tests with just the 539 breast cancer patients (excluding 29 ovarian cancer patients) yielded almost similar results, except for *ATM*, which was no longer significant (OR = 11.3, 10.8, 3.8, and 2.4, respectively; *p* < 0.01, *p* < 0.01, *p* = 0.03, and *p* = 0.07, respectively).

The inclusion of male data in the case-control analysis above may have under- or over-estimated the ORs for the genes of interest. Therefore, we performed another case-control analysis for Japanese female HBOC data (*n* = 566; data for 2 male patients were excluded) as compared with female ExAC data (excluding TCGA; *n* = 22,937) without a distinction of ethnicity (Table 2b)^13^. For this comparison, data from the HGVD and TMM databases were not included in the controls, as they lacked allele count information for gender. We still observed significant enrichments in *PALB2*, *BARD1*, *BLM*, and *ATM* (Table 2b)^13^. Furthermore, excluding the “ovarian-only” patients from the Japanese HBOC cohort did not significantly change the results (OR = 9.4, 15.6, 3.6, and 3.2; *p* < 0.01, *p* < 0.01, *p* = 0.03 and *p* = 0.03, respectively), indicating robustness of the enrichment in the Japanese HBOC cases. These observations suggest the relevance of *PALB2*, *BARD1*, and *BLM* in breast cancer susceptibility in Japanese HBOC patients.

### Prevalence of mutated genes between Japanese and previous cohorts of familial breast cancer

**Fig. 3:**
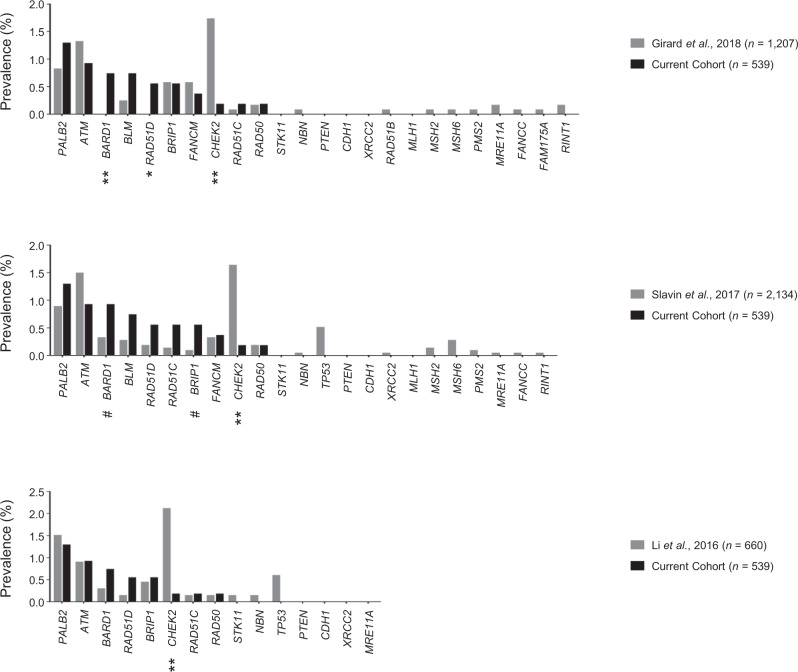
Prevalence of mutated genes in previous and current familial breast cancer cohorts. Top panel. Prevalence of mutated genes compared with those in the French cohort^21^. Prevalence (%) is shown as bar plots. Gray and black denote the French and current Japanese cohorts, respectively. Mutations in the graph are truncating SNVs/indels. The prevalence of *TP53* mutations is not included because pathogenicity information for the missense variants was not available. Middle panel. The US cohort (gray). Mutations in the graph are SNVs, indels, and CNVs. Bottom panel. Prevalence of mutated genes compared with those in the Australian cohort (gray)^23^. Mutations are SNVs and indels. Genes are aligned according to their prevalence in the Japanese cohort. *P*-values were computed using Fisher exact tests based on the presence or absence of a mutation between the cohorts, and are shown below the gene symbols when significant or near significant. *p < 0.05, **p < 0.01, and #*p* < 0.1.

*BARD1* and *BLM* are not typically included in top prevalent gene lists for Caucasian-dominant populations^16–22^. To further explore the potential ethnic differences in the distribution of disease-causing genes, we compared the mutational frequencies between the current and previous familial breast cancer (FBC) cohorts. Few previous studies have had sufficient sample size (*n* ≥ 500) to ascertain mutational prevalence based on family history^20,21,23–25^. We selected three datasets (Australian, US, and French cohorts) that were presumably derived from Caucasian-dominant populations. These studies provided detailed family histories and sufficient information to re-calculate prevalence^20,21,23^. We compared the mutational frequencies in each cohort separately because of differences in study design: number of common target genes (15, 23, and 24 for the Australian, US, and French studies, respectively), the presence/absence of CNV data (missing in the Australian and French studies), and the presence/absence of missense variant data (missing in the French study). Using Fisher exact tests, we detected a significantly less frequent distribution of *CHEK2* mutation in the Japanese cohort than in the Caucasian-dominant cohorts (OR = 0.1, 0.1 and 0.1 for the French, US and Australian cohorts, respectively; all *p* < 0.01). In contrast, the Japanese cohort had more frequent mutations in *BARD1* and *RAD51D* (both ORs were infinite; *p* < 0.01 and *p* = 0.03) as compared with the French cohort, and there was a consistent upward trend (*p* > 0.05) for these differences when compared with the US and Australian cohorts. Similarly, the *BLM* mutation seemed more enriched in the Japanese than in the French and US cohorts but this difference was not significant (Fig. 3)^13^. These findings implicate ethnicity-related differences in the distribution of mutated genes among HBOC patients.

### Patient and tumor characteristics of P/LP carriers

Patient characteristics and tumor features for subjects with P/LP variants are provided in Tables 3 and 4, and Fig. 2^13^. All 37 *BRCA1/2* WT cases with 38 P or LP variants (35 SNVs/indels and 3 CNVs) had strong family history (HBOC history level 1 or 2; see Methods and Supplementary Note 1), and data were available regarding primary tumor site (breast or ovary), age at primary tumor diagnosis, breast cancer laterality, and other cancer type (Table 3 and Fig. 2)^13^. We found that patients with germline *RAD51D* mutations were diagnosed with cancer at a significantly younger age than patients with other mutations (*p* = 0.01 by Mann–Whitney *U*-test; Table 3)^13^. No other associations with patient characteristics were observed (Table 3)^13^.

**Table 3:** Patient characteristics with germline pathogenic variants in 28 disease-causing genes (excluding *BRCA1/BRCA2*).

Case	Variant	Patient characteristics				
		HBOC history level	Primary tumor site	Age at primary tumor diagnosis	Breast cancer laterality	Additional cancer
A1013	*PALB2*:p.M723fs	1	Breast	36	Unilateral (*n* = 1)	–
A0139	*PALB2*:p.R1086*	1	Breast	63	Unilateral (*n* = 1)	–
B0215	*PALB2*:p.R1086*	2	Breast	37	Unilateral (*n* = 1)	–
A0214	*PALB2*:p.E462*	2	Breast	50	Bilateral (*n* ≥ 2)	–
B0219	*PALB2*:p.E462*	1	Breast	54	Bilateral (*n* ≥ 2)	Thyroid
B0277	*PALB2*:p.L484*	2	Breast	47	Unilateral (*n* = 1)	–
A0338	*PALB2*:p.T274fs	2	Breast	44	Bilateral (*n* ≥ 2)	–
D0241	*ATM*:p.R805*	2	Ovary	50	Not applicable	–
E0125	*ATM*:p.P80fs	2	Breast	47	Unilateral (*n* = 1)	–
A0346	*ATM*:p.Q374fs	1	Breast	65	Unilateral (*n* *=* *1)*	–
B0242	*ATM*:c.4776+2T>A: Splice Site	2	Breast	49	Unilateral (*n* = 1)	–
C0158	*ATM*:p.F1837fs	2	Breast	48	Unilateral (*n* = 1)	–
A0301	*BARD1*:p.R641*	2	Breast	58	Unilateral (*n* = 1)	–
A0331	*BARD1*:p.R150*	2	Breast	26	Unilateral (*n* = 1)	–
	*ATM*:p.A2626fs	2	Breast	26	Unilateral (*n* = 1)	–
A0298	*BARD1*:p.Q449*	1	Breast	35	Unilateral (*n* = 1)	–
B0258	*BARD1*:p.A173fs	2	Breast	42	Bilateral (*n* ≥ 2)	–
E0114	*BARD1*:Exon 5–7 Deletion	1	Breast	42	Bilateral (*n* ≥ 2)	Endometrial
C0120	*RAD51D*:p.K111fs	1	Breast	33	Unilateral (*n* = 1)	–
D0239	*RAD51D*:p.K111fs	2	Ovary	58	Not applicable	–
A0231	*RAD51D*:p.V152fs	1	Breast	40	Bilateral (*n* ≥ 2)	–
B0220	*RAD51D*:p.Q149*	2	Breast	39	Unilateral (*n* = 1)	–
D0221	*BLM*:p.S106fs	1	Breast	40	Unilateral (*n* = 1)	–
C0256	*BLM*:p.S106fs	1	Breast	49	Bilateral (*n* ≥ 2)	–
B0187	*BLM*:p.G512fs	1	Breast	49	Unilateral (*n* = 1)	–
B0292	*BLM*:c.3751 + 2 T > C: Splice Site	2	Breast	49	Unilateral (*n* = 1)	–
A0281	*BRIP1*:p.R356*	1	Breast	33	Unilateral (*n* = 1)	–
B0285	*BRIP1*:p.A1081fs	1	Breast	47	Bilateral (*n* ≥ 2)	–
B0170	*BRIP1*:c.918 + 2 T > C: Splice Site	1	Breast	41	Unilateral (*n* ≥ 2)	Thyroid
E0237	*RAD51C*:c.G133T:p.E45*	2	Breast	50	Unilateral (*n* = 1)	–
C0206	*RAD51C*:Exon 6–9 Deletion	2	Breast	51	Unilateral (*n* = 1)	–
B0263	*RAD51C*:Exon 6–9 Deletion	2	Breast	45	Bilateral (*n* ≥ 2)	–
B0288	*FANCM*:p.Q730fs	2	Breast	34	Unilateral (*n* = 1)	–
D0231	*FANCM*:p.K841fs	2	Breast	41	Unilateral (*n* = 1)	–
E0131	*RAD50*:p.D544fs	2	Breast	49	Unilateral (*n* = 1)	–
B0198	*NF1*:p.G255fs	1	Breast	25	Unilateral (*n* = 1)	–
A0277	*CHEK2*:p.L486fs	1	Breast	37	Unilateral (*n* = 1)	–
D0222	*RECQL*:p.D517_S518delins*	2	Breast	57	Unilateral (*n* = 1)	–

Results are for 35 of 37 cases with breast cancer (2 patients [*ATM* p.R805* and *RAD51D* p.K111fs] with ovarian cancer only were excluded). Unilateral (*n* = 1), Unilateral (*n* ≥ 2) and Bilateral (*n* ≥ 2) indicate one unilateral occurrence, at least two unilateral occurrences, and at least two bilateral occurrences of primary breast cancer, respectively. The tumors were defined as independent primaries (not local recurrent tumors) when cancer cells were absent in the surgical margin of the first tumor, and also when the difference could be seen in the position of occurrence, histology, hormonal status and HER2 expression of the second tumor, according to previous criteria^50^.

**Table 4:** Breast cancer properties in patients with germline pathogenic variants in 28 disease-causing genes.

Case	Variant	Breast cancer properties					
		Tumor ID	Age at surgery	Histology	Hormonal subtype	Nuclear grade	Loss of wildtype allele
A1013	*PALB2*:p.M723fs	T1	36	Solid Tubular	Luminal	1	NA
A0139	*PALB2*:p.R1086*	T1	63	Scirrhous	Luminal	3	AST
B0215	*PALB2*:p.R1086*	T1	37	Solid Tubular	Luminal-HER2	3	NA
A0214	*PALB2*: p.E462*	T1	50	Scirrhous	Luminal	NA	AST
		T2	55	Scirrhous	Luminal-HER2	1	NA
B0219	*PALB2*:p.E462*	T1	54	Solid Tubular	Luminal	2	AST
		T2	58	Non-invasive Ductal	Triple Negative	1	LOH
		T3	61	Solid Tubular	Triple Negative	3	NA
B0277	*PALB2*:p.L484*	T1	47	Scirrhous	Triple Negative	3	AST
A0338	*PALB2*:p.T274fs	T1	44	Scirrhous	Luminal	3	NA
		T2	44	Scirrhous	Luminal	3	NA
E0125	*ATM*:p.P80fs	T1	47	Mucinous	Luminal	NA	LOH
A0346	*ATM*:p.Q374fs	T1	65	Scirrhous	Luminal	1	NA
B0242	*ATM*:c.4776+2T>A	T1	49	Scirrhous	Luminal	2	ND
C0158	*ATM*:p.F1837fs	T1	48	Solid Tubular	Luminal	2	LOH
A0301	*BARD1*:p.R641*	T1	58	Solid Tubular	Triple Negative	3	NA
A0331	*BARD1*:p.R150*	T1	26	Scirrhous	Luminal	3	NA
	*ATM*:p.A2626fs	T2					
A0298	*BARD1*:p.Q449*	T1	35	Scirrhous	Triple Negative	3	NA
B0258	*BARD1*:p.A173fs	T1	42	Solid Tubular	Triple Negative	3	LOH
		T2	57	Solid Tubular	Triple Negative	3	NA
E0114	*BARD1*:Exon 5–7 Deletion	T1	42	Papillotubular	Luminal (Unk. HER2)	1	NA
		T2	51	Tubular	Luminal (Unk. HER2)	1	NA
		T3	64	Scirrhous	NA	2	ND
C0120	*RAD51D*:p.K111fs	T1	33	Papillotubular	Luminal	3	LOH
A0231	*RAD51D*:p.V152fs	T1	40	Non-invasive Ductal	Luminal (Unk. HER2)	2	ND
		T2	47	Non-invasive Ductal	Luminal	1	NA
B0220	*RAD51D*: p.Q149*	T1	39	Scirrhous	Luminal	2	ND
D0221	*BLM*:p.S106fs	T1	40	Non-invasive Ductal	Luminal (Unk. HER2)	NA	NA
C0256	*BLM*: p.S106fs	T1	49	Scirrhous	Luminal	2	NA
		T2	49	Solid Tubular	Luminal	2	NA
B0187	*BLM*: p.G512fs	T1	49	Solid Tubular	Luminal	1	ND
B0292	*BLM*:c.3751+2T>C	T1	49	Scirrhous	Luminal	1	NA
A0281	*BRIP1*:p.R356*	T1	33	Mucinous	Luminal	1	NA
B0285	*BRIP1*:p.A1081fs	T1	47	Non-invasive Ductal	Luminal	1	LOH
		T2	47	Papillotubular	Luminal	1	NA
B0170	*BRIP1*:c.918+2T>C	T1	41	Scirrhous	Luminal	1	NA
		T2	41	Non-invasive Ductal	Luminal (Unk. HER2)	NA	ND
E0237	*RAD51C*:p.E45*	T1	50	Scirrhous	Luminal	2	NA
C0206	*RAD51C*:Exon 6–9 Deletion	T1	51	Scirrhous	Triple Negative	2	LOH
B0263	*RAD51C*:Exon 6–9 Deletion	T1	45	Scirrhous	Luminal (Unk. HER2)	NA	NA
		T2	45	Non-invasive Ductal	NA	NA	ND
B0288	*FANCM*:p.Q730fs	T1	34	Solid Tubular	Triple Negative	3	ND
D0231	*FANCM*:p.K841fs	T1	41	Non-invasive Ductal	Luminal	1	NA
E0131	*RAD50*:p.D544fs	T1	49	Scirrhous	Luminal-HER2	3	ND
B0198	*NF1*:p.G255fs	T1	25	Solid Tubular	Luminal	2	LOH
A0277	*CHEK2*: p.L486fs	T1	37	Papillotubular	Luminal	1	ND
D0222	*RECQL*:p.D517_S518delins*	T1	57	Solid Tubular	NA	NA	NA

Results are for 48 primary breast tumors from 35 cases with breast cancer (2 patients [*ATM* p.R805* and *RAD51D* p.K111fs] with ovarian cancer only were excluded).

T1, T2, and T3 indicate the first, second and third primary breast tumors from the same patient.

*Unk. HER2* unknown status for HER2, *NA* information or assay was not available for the tumor, *LOH* loss of heterozygosity by copy number (CN) loss, *AST* additional somatic truncation, *ND* not detected.

We next sought to examine genotype–phenotype correlations among those with breast cancer. Thirty-five patients had 47 breast carcinomas, and data pertaining to the age at surgery and the type of histology were known. Data for hormonal status and nuclear grade were also available for most of these carcinomas (45/47 and 41/47, respectively; Table 4 and Fig. 2)^13^. Among patients with breast tumors, *BARD1* mutations were correlated with solid tubular histology, nuclear grade 3, and triple-negative subtype (OR = 6.7, 10.0, and 6.0, respectively; *p* = 0.01, *p* < 0.01, and *p* = 0.02, Fisher exact tests; Table 4)^13^. Similarly, germline *PALB2* mutations were associated with solid tubular histology and nuclear grade 3 (OR = 3.8 and 5.4; *p* = 0.01 and *p* = 0.02, respectively; Fisher exact tests; Table 4)^13^. Consistent with previous observations^26,27^, these findings show that genotype–phenotype correlations were well captured in the current study. Furthermore, we sought to identify factors that could possibly distinguish between patients with a non-*BRCA1/2* pathogenic variant and those without such a mutation. However we only detected younger onset as an associated but not a predictive factor of these mutations (Supplementary Note 3).

### Somatic mutation analyses in tumor samples

Twenty-two of the 47 breast carcinomas were available for targeted re-sequencing (Supplementary Methods, Tables 4 and 5)^13,28^. LOH or AST mutation was detected in at least one tumor with *PALB2*, *ATM*, *RAD51D*, *BARD1*, *BRIP1*, *RAD51C*, or *NF1* germline mutation. The results of tumor analyses were confirmatory for *PALB2* (5 ASTs in 5 tumor samples) and supportive for *ATM* (2 LOHs in 3 tumors), but the other genes contributed less (limited analyses). Nevertheless, these additional somatic events implicate the pathogenicity of germline variants as driver events in tumorigenesis (Tables 4 and 5)^13^. Of note, four ASTs were detected for the P/LP variants, all on *PALB2* (c.3114–1 G > A [splice site SNV], p.N455fs, p.Y28fs and p.E218fs), and significantly frequently co-occurred with germline *PALB2* mutant breast cancers (OR = 776.0; *p* < 0.01, Fisher exact test; Tables 4 and 5)^13^. In total, 8 and 4 tumors exhibited LOH and AST, respectively, on the mutated genes (Table 5)^13^. Comparisons were also made between the presence/absence of LOH/AST in one of the 28 causal genes with a P/LP variant and those with a VUS variant, an LB/B variant, or any of the other 89 gene variants. We detected a significantly frequent loss of wildtype allele events in genes with pathogenic variants (OR = 7.2, 4.8, and 7.5, respectively; all *p* < 0.01, Fisher exact test; Table 5), validating the pathogenicity interpretations performed in the current study.

**Table 5:** Summary of loss of heterozygosity and additional somatic truncating mutations detected in genes.

Gene	Number of germline mutant alleles	Number of tumor analyzed	LOH	AST	ND	Ratio of LOH or AST
*PALB2*	7	5	1	4	0	5/5 (100%)
*ATM*	6	3	2	0	1	2/3 (67%)
*RAD51D*	4	3	1	0	2	1/3 (33%)
*BARD1*	5	2	1	0	1	1/2 (50%)
*BRIP1*	3	2	1	0	1	1/2 (50%)
*RAD51C*	3	2	1	0	1	1/2 (50%)
*NF1*	1	1	1	0	0	1/1 (100%)
*BLM*	4	1	0	0	1	0/1 (0%)
*FANCM*	2	1	0	0	1	0/1 (0%)
*RAD50*	1	1	0	0	1	0/1 (0%)
*CHEK2*	1	1	0	0	1	0/1 (0%)
*RECQL*	1	0	0	0	0	0/0 (NA)
Total	38	22	8	4	10	12/22 (54.5%)

*LOH* loss of heterozygosity by copy number (CN) loss, *AST* additional somatic truncation, *ND* not detected, *NA* not available.

### Family member analysis

Germline samples for 34 members among 13 families were subjected to targeted re-sequencing for the 28 disease-causing genes (Supplementary Methods). Briefly, in 4 families, the mutant and wildtype alleles showed exact concordance with breast cancer occurrence for variants including *PALB2* and *BLM*, but the remaining 9 families did not perfectly match with the presence of breast or ovarian disease (Supplementary Note 4; Supplementary Fig. 6).

## Discussion

Numerous studies have assessed the prevalence of breast cancer susceptibility genes not only among unselected, consecutive patients but among specific patient cohorts^1,2,29,30^. For subjects of east-Asian ethnicity, only two studies have had sufficient sample size of unselected, consecutive Chinese (*n* = 8085)^7^ or Japanese (*n* = 7051)^31^ breast cancer patients for analysis. Likewise, FBC cohorts tend to have too few numbers^20,21,23–25^. In the current study, we performed next-generation sequencing analyses on 28 previously known disease-causing genes for HBOC syndrome, focusing on 568 Japanese *BRCA1/2* WT index patients with a strong family history. Through our analysis, we demonstrate the non-negligible impact of non-*BRCA1/2* mutations in Japanese patients with HBOC syndrome, with the following rates of germline mutations: 8.5% *BRCA1*, 10.1% *BRCA2*, 0.2% *BRCA1*/*2*, and 6.5% for non-*BRCA1/2* genes (5 cases had recurrent variants among 37 patients with non-*BRCA1/2* mutations). These figures clearly demonstrate an emergent need to incorporate genes other than *BRCA1/2* for precision preventive medical care. Although it is difficult to directly compare rates of non-*BRCA1/2* mutations across different studies due to differences in ethnicity and target gene selection, our rates are more or less comparable with those in previous *BRCA1/2*-WT FBC cohorts^20,23^, implying a similar clinical impact of these genes.

In our case-control analyses with a non-cancer east-Asian population and non-cancer females, we found significant prevalence of *PALB2*, *BARD1*, *BLM*, and *ATM* mutations in our HBOC cohort, including patients with ovarian cancer. These results are inconsistent with previous observations in European-dominant cohorts, where *PALB2*, *CHEK2*, and *ATM* mutations are typically detected as moderate-risk genes^16,17,20,21,23–25^. *CHEK2* c.1100delC:p.T367fs is one of the most prevalent pathogenic variants among those of European ancestry^32^; yet, *CHEK2* mutations were rare among our Japanese cohort. While this difference may simply reflect a low frequency of *CHEK2* mutations in unselected, consecutive patients with breast cancer, previous Chinese and Japanese studies also failed to detect *CHEK2* c.1100delC, and showed a low prevalence of other *CHEK2* mutations^7,16,17,31^.

*BARD1* was significantly enriched in both the case-control analyses (non-cancer east-Asian population and with non-cancer females) and against the French cohort. *RAD51D*^21^ was also enriched against the French cohort. These findings may suggest an ethnic association for these two genes. In previous unselected patient studies, *RAD51D* mutations were highly prevalent in Chinese-dominant versus Caucasian-dominant populations, supporting possible *RAD51D* enrichment among east-Asians^7,16,17^. *BARD1*, on the other hand, was significantly enriched in a US FBC cohort^20^, supporting its relevance at least as a breast cancer susceptibility gene.

In the current cohort, *BLM* c.319dupT p.S106fs was detected in 2 cases: this mutation has not been described previously for Japanese persons with Bloom syndrome. There are six Japanese patients in the Bloom Syndrome Registry^33,34^, five of whom are homozygous/transheterozygous for the *BLM* c.557_559delCAA p.S186* variant^33,34^, a variant not found among our cohort. Comparatively, among 14 Chinese patients with breast cancer bearing *BLM* deleterious variants^7^, three patients carried the *BLM* c.319dupT p.S106fs variant that we detected but none had the previous *BLM* c.557_559delCAA p.S186* variant. Despite the small sample sizes, our observations point to a possible biological difference between *BLM* variants. Moreover, *BLM* enrichment in the case-control analyses was partially supported by exact concordance of the variant with affected family members in one family; however, enrichment was not confirmed against previously studied cohorts or in the tumor analysis, and more data are needed to confirm if these enrichments are associated with ethnicity.

Moreover, our family member analyses point to the need for further study to avoid overlooking important yet concealed genetic links within families predisposed to HBOC syndrome. Although a mutant gene was not necessarily shared by family members with breast or ovarian cancer (e.g., affected sisters had different gene mutations), an unaffected member or member with another type of cancer often retained the same mutation as the index patient. Such gene complexity is frequently noted^23,35^ and may reflect the low penetrance of genes with multiple individual gene involvement in HBOC syndrome. These discrepancies highlight the need to test a panel of genes, not just a single site on a gene of interest.

In the case-control analyses, *NF1* mutation was less enriched in the current cohort. This is perhaps because patients with type-1 neurofibromatosis (NF1) develop clinical manifestations by the age of 10 years (cafe-au-lait macules, skin tumors and scoliosis^36^), which is much earlier than the typical age of onset of *NF1*-associated breast cancers^37^. Since most symptomatic patients are tested for *NF1* mutations before they visit a breast cancer clinic, these patients are not tested for mutations in *BRCA1/2* genes for their breast disease, a requisite for enrollment in the current study.

Limited data availability created several shortcomings in our evaluations of the two case-control analyses. For instance, ExAC provides allele count data for gender and ethnicity separately, but not combined. Moreover, neither HGVD nor TMM provides allele count information for gender. As such, the use of similar ethnicity data as the control does not allow us to further filter allele count data based on gender, and vice versa. In the first case-control comparison, the control comprised metadata of non-cancer east-Asian population from HGVD, TMM, and east-Asian non-TCGA ExAC datasets. Each dataset included male data and was generated with different ascertainment from each other as well as from the current cohort. In the second case-control analysis, data were non-TCGA female ExAC data predominantly derived from a Caucasian population. Also worth noting, although subjects were not suffering from cancer at the time of germline sample collection, this does not ensure a life-long cancer-free condition. Furthermore, different sequencing methodologies and informatics analyses may affect variant detection. These limitations may lead to an under- or over-estimation of the ORs. As such, any correlations should be carefully interpreted.

LOH and AST on the wildtype allele completely inactivate the function of a gene with a germline heterozygous loss-of-function mutation^6,38–40^. Although the number of tumor analyses was limited in the current study, our detection of LOH and AST in a significant proportion of tumor samples with pathogenic variants strongly supports the pathogenicity of the germline mutations during breast cancer development. LOH or AST has been detected in numerous previous studies investigating *BRCA1*/*BRCA2* alleles (over 80% frequency)^6,38–40^, and for *ATM* and *PALB2* (<50% of tumor samples)^41–44^, similar to our findings. We further found that tumors with *BARD1* p.A173fs and *RAD51D* p.K111fs germline mutations exhibited LOH, supporting the pathogenicity of these variants. The roles of the mutated genes in tumors without LOH or AST remain unknown; these tumors might develop via gene haplo-insufficiency, or the gene may have no critical role. Indeed, the frequency of LOH and AST might simply reflect the relatively low penetrance of moderate-risk genes. Alternatively, wildtype allelic inactivation could be accomplished by epigenetic gene silencing with DNA hypermethylation; albeit, promoter methylation is reported to contribute little to *BRCA1* wildtype allelic inactivation^45^. The specific detection of AST in *PALB2*-mutant tumors may indicate that *PALB2* structure favors AST as an inactivating mechanism of the wildtype allele.

In conclusion, we detected a significantly high prevalence of *PALB2*, *BARD1*, and *BLM* mutations, with a low frequency of mutant *CHEK2* in the current Japanese cohort of 568 patients with *BRCA1/2* WT HBOC syndrome. We confirmed associations of *BARD1* and *PALB2* with the triple-negative subtype and ASTs, and found significant loss-of-function mutations on the wildtype allele of genes with germline mutations in the tumor samples. Whereas *BARD1*, *BLM*, and *RAD51D* mutations have a possible ethnic association, we identified only partial support for tumor and family member associations due to the limited sample size. Nevertheless, the current study provides a solid basis to provide medical care to Japanese patients with HBOC syndrome.

## Methods

### Study structure

Patients included in the study were from six academic and cancer hospitals in Japan: Showa University Hospital (Hatanodai, Tokyo), Cancer Institute Hospital (Ariake, Tokyo), St. Luke’s International Hospital (Akashi-cho, Tokyo), Shikoku Cancer Center (Minami-umemoto-cho, Matsuyama), Sagara Hospital (Matsubara-cho, Kagoshima), and Keio University Hospital (Shinano-cho, Tokyo). These institutions participated in the “Project for Development of Innovative Research on Cancer Therapeutics” (P-DIRECT; 2014–2015) research program, and in the succeeding “Project for Cancer Research and Therapeutic Evolution” (P-CREATE; 2016–2017) program, granted by the Japan Agency for Medical Research and Development (AMED).

### Ethics approval and consent to participate

Sample acquisition and genetic analyses were approved by the institutional review board at each institution. After genetic counseling, written informed consent was obtained from all participants (probands or family members).

### Patient groups in the current study

Two groups of patients diagnosed with HBOC syndrome were enrolled in the current study: 1) the first group comprised patients who were negative for a *BRCA1/2* genetic test (*n* = 230); and 2) the second group comprised patients who had not yet been tested for *BRCA1/2* mutations (*n* = 436) (Fig. 1). *BRCA1/2* genetic testing for the second group was performed as described below. Germline DNA from a total of 568 *BRCA1/BRCA2* WT, 27 *BRCA1/BRCA2* mutation*-*positive and 10 VUS cases were rendered to exome sequencing analyses (Fig. 1). A panel of 119 genes (Supplementary Methods and Supplementary Table 1) was designed and used to validate the detected variants by exome sequencing using the same germline DNA, and to assess the mutational status of the tumor and family member samples^13^.

### Eligibility criteria

In the current study, 2 levels of criteria were used to determine eligible patients based on the prevalence of breast or ovarian cancer among family members, as originally described by Nomizu^46^, with slight modifications. HBOC history level 1 corresponds to an individual with a breast or ovarian cancer diagnosis, who meets any of the following: (1) two or more first-degree relatives suffered from breast or ovarian cancer; or (2) one or more first-degree relative suffered from breast or ovarian cancer that: (2-a) was diagnosed before the age of 40 years, (2-b) arose as a part of synchronous or asynchronous bilateral primary breast cancer, and/or (2-c) arose as a part of synchronous or asynchronous multiple primary cancer. Level 2 corresponds to an individual with a breast or ovarian cancer diagnosis who had one or more first- or second-degree relatives who suffered from breast or ovarian cancer (Supplementary Note 1, Supplementary Fig. 1a).

### *BRCA1/2* mutation test and clinicopathological information

*BRCA1/2* genetic testing was performed at Falco Biosystems (Shimizu-cho, Kyoto) using the method licensed by Myriad Genetics (Salt Lake City, Utah) between 2014 and 2016, or at Ambry Genetics (Aliso Viejo, California) using the OvaNext 25-gene panel in 2017. Multiplex ligation-dependent probe amplification (MLPA) analysis was performed for all patient samples^47^. Data on *BRCA1/2* and the clinicopathological information of the participants were registered with the Japanese HBOC consortium database center located at Showa University (Hatanodai, Tokyo)^48^.

### Independent primaries

Using previous criteria^49^, we defined tumors as independent primaries (but not local recurrent tumors) when there was an absence of cancer cells in the margin of the first tumor, and when a difference could be seen in the position of occurrence, histology, hormonal status, and HER2 expression of the second tumor.

### Sample acquisition

Blood was collected from probands, and saliva or blood was provided by family members. Twelve fresh-frozen and 163 formalin-fixed paraffin-embedded (FFPE) tumor samples were obtained through biopsy or surgical specimens.

### Sample preparation for sequencing

Frozen or FFPE tissues were cut into 10-μm-thick sections. The selective enrichment of cancer cells was performed by manual macrodissection or laser-capture microdissection with an LMD7000 (Leica) following the manufacturer’s protocol. DNA from whole blood, saliva, fresh-frozen, and FFPE tumors was extracted using the QIAamp DNA Blood Mini Kit (Qiagen), Oragene DNA Kit (DNA Genotek), QIAamp DNA Micro Kit (Qiagen), and the GeneRead DNA FFPE Kit (Qiagen), respectively. DNA quality and quantity were checked with a NanoDrop 2000 (Thermo Fisher Scientific) and Qubit 2.0 fluorometer (Thermo Fisher Scientific). DNA samples that passed the criteria for DNA purity (optical density 260/280 nm >1.8), ratio of dsDNA/ssDNA concentration (>0.35), and dsDNA concentration (>50 ng/μl) were further processed to exome or panel sequencing.

### Terminologies used for 5-tier and 3-tier pathogenicity descriptions

A 5-tier system is used by commercial companies and the ACMG-AMP (American College of Medical Genetics and Genomics-Association for Molecular Pathology) guidelines to express the pathogenicity of variants, as follows: “deleterious” (or “pathogenic”), “likely deleterious” (or “likely pathogenic”), “uncertain significance”, “favor polymorphism” (or “likely benign”) and “polymorphism” (or “benign”). However, where necessary, we used a 3-tier system, with “deleterious/pathogenic” and “likely deleterious/likely pathogenic” as “mutated” or “pathogenic”, and “favor polymorphism/likely benign” and “polymorphism/benign” as “wildtype” or “benign”.

### Library preparation and sequencing for exome analysis

The sequencing method for exome and target panel analyses has been described previously^50^. The median coverage was 123 reads per germline exome, 366 reads per germline target panel, and 790 reads per tumor target panel sequencing. A total of 568 proband and 34 family member germline specimens, and 11 fresh-frozen and 146 FFPE tumor samples finally passed the stringent quality assessments during sample preparation, sequencing, and informatics analyses for targeted re-sequencing or exome sequencing.

### Germline-variant analysis

Sequenced reads were aligned with BWA (Burrows-Wheeler Aligner; ver. 0.6.1) to the reference human genome (hg19)^51^. GATK (GenomeAnalysisTK; ver. 3.4–46) was used to recalibrate variant quality scores and to perform local realignment^52^. Germline variants were called with GATK UnifiedGenotyper and HaplotypeCaller (GATK ver. 3.4–0)^14^ and considered as genuine when detected by both software.

Germline variants were taken as significant with the following conditions: (1) SNVs or in-frame or frame-shift indels in coding exons, or splice-site variants (±2 bp at the exon-intron boundary); (2) variants with a read depth ≥20; (3) variants with a read frequency ≥0.2; (4) variants with a global minor allele frequency (MAF) score <0.01 in ExAC (ver. 0.3.1, with TCGA data removed), NHLBI Exome Sequencing Project (ESP6500; ver. ESP6500SI-V2) or 1000 Genomes Project (Phase 3). Germline CNVs were detected with eXome-Hidden Markov Model (XHMM; ver. 1.0)^53^. We took CNVs detected by XHMM analysis of the exome data as genuine after validation using an Affymetrix Genome-Wide Human SNP Array 6.0. CNVs detected by the array were called with Genotyping Console version 4.2.0.26. The MutationMapper tool from cBioPortal (http://cbioportal.org)^54,55^ was used to annotate germline variants in a gene in the lollipop-style mutation diagram.

### Population databases

To compare the MAF in our cohort with that in population databases for Japanese people, we used the HGVD (Human Genetic Variation Database; ver. 1.42)^56^ or TMM (Tohoku Medical Megabank Project; hum0015.v1)^57,58^. HGVD (*n* = 1,208) and TMM (*n* = 3554) comprise only Japanese persons without major diseases, including cancer^56–58^. ExAC (ver. 0.3.1) without 7601 TCGA data were used as the control. ExAC data were split into 3933 “East-Asian” and 49,261 “Other ethnicity” for subsequent analyses^59^. Three population databases (HGVD, TMM and non-TCGA ExAC) contained allele count information for SNVs/indels from a population without cancer; CNV allele count information was not available in the HGVD or TMM databases.

### Disease variant databases

Reported interpretations and some additional information, such as reference literature for known variants, were obtained through the HGMD (ver. 2017.2) and ClinVar (4/May/2017).

### Somatic SNV/indel/CNV

Somatic SNVs were called with VarScan (ver. 2.3.7)^60^, MuTect (ver.1.1.4)^61^, and Karkinos (ver. 3.0.22) (http://sourceforge.net/projects/karkinos/). VarScan (ver. 2.3.7), SomaticIndelDetector (ver.1.5–30)^62^, and Karkinos (ver.3.0.22) were used to detect somatic indels. Somatic SNVs and indels were taken as genuine mutations when they were detected with at least two among three callers. When necessary, somatic CNVs were detected by EXCAVATOR (ver. 2.2)^63^. Whereas EXCAVATOR requires sufficient number distribution of probes on chromosome arms, probes for the 119-gene panel are not sufficient; therefore, we used exome sequencing instead of the panel for this purpose. For germline SNVs/indels, LOH of the WT allele by copy number loss was determined when the variant read frequency was between 0.2 and 0.6 for the germline DNA and more than 0.6 for the tumor samples^64^. For germline CNVs, LOH (additional copy number loss) was called by a decrease in the log-2 ratio; the log-transformed ratio of the exon mean read count between tumor and germline samples was normalized with the LOWESS scatter plot normalization procedure.

### Pipeline based on the algorithm per the ACMG-AMP guidelines

For the pathogenicity classification in our study, we constructed a pipeline (Supplementary Note 2 and Supplementary Fig. 3) based on an algorithm according to the ACMG-AMP guidelines, using methodologies as described previously^65,66^. Among 27 codes to determine the pathogenic or benign impact of a variant (PVS1, PS1–PS4, PM1–PM6, PP1–PP5, BP1–BP6, BS1–BS4, and BA1), we did not employ 9 codes (PS2, PM3, PM6, PP1, PP4, BS2, BS4, BP2, and BP5) for the following reasons: (1) we did not presume the presence of any de novo variant in the current study, because the subjects were patients with a family history (PS2 and PM6); (2) HBOC syndrome is not a monogenic disease (PP4); (3) frequent variants were already filtered (MAF ≥ 0.01) before pathogenicity classification (BS2); and (4) the same variants should be classified into a same pathogenic category to achieve equitable evaluation in several analyses, such as the occurrence of LOH in the tumor. The following codes might produce a different pathogenicity assignment to a variant (co-occurrence of clear causative variant in a patient; BP5, phasing two variants in a gene; PM3 and BP2, and segregation with family member genetic information; PP1 and BS4). Eighteen attributes were finally employed for the raw calls (Supplementary Fig. 3). Manual inspections were conducted when the raw calls were (1) discordant with the locus-specific databases or ClinVar, (2) classified as LP or P, or (3) derived from truncating variants^66^.

### Case-control analysis

Case-control analyses were performed as previously described^20^. Two-sided Fisher exact tests with R (ver. 3.3.1) were used to compute ORs between the Japanese HBOC cases and the controls. Allele counts for SNVs/indels were summed for each gene. The total allele count for the controls was calculated with the maximal number of the highest quality allele calls across exonic regions.

Because ExAC does not provide data for gender and ethnicity combined, and because neither HGVD nor TMM provides allele count information for gender, we performed two different case-control analyses with each of the control data: (1) HGVD, TMM, and east-Asian data of the ExAC were combined as metadata (*n* = 8695) as the control, and (2) female ExAC without distinction of ethnicity (*n* = 22,937; excluding TCGA) was used as the control.

### Other statistical analyses

Mann–Whitney *U*-test, Fisher’s exact test and logistic regression analyses were used to statistically evaluate the correlation between clinicopathological parameters and pathogenicity classifications using GraphPad Prism (ver. 8.0.2) or R (ver. 3.3.1) software.

### Reporting summary

Further information on research design is available in the Nature Research Reporting Summary linked to this article.

## Supplementary information

Supplementary

Reporting Summary Checklist

## Data Availability

The datasets supporting Figs. 2, 3, Tables 1–5 and supplementary table 1, are publicly available in the figshare repository here: 10.6084/m9.figshare.11959233^13^. Germline SNV/indel data are publicly available in ClinVar under the following ClinVar submission accession IDs: SCV001193462 – SCV001193759. The 298 ClinVar submissions can also be accessed here: https://www.ncbi.nlm.nih.gov/clinvar/submitters/507256/^15^. Targeted re-sequencing data are available in the National Bioscience Database Centre (NBDC) under the dataset accession ID JGAS00000000224^28^. Exome sequencing data are not publicly available in order to protect patient privacy, but can be accessed from the corresponding author, Dr. Seigo Nakamura (email: seigonak@med.showa-u.ac.jp), on reasonable request.

## Code availability

The code for pathogenicity classification is written in Python and available upon request.

## References

[1] Hilbers FS, Vreeswijk MP, van Asperen CJ, Devilee P (2013). The impact of next generation sequencing on the analysis of breast cancer susceptibility: a role for extremely rare genetic variation?. Clin. Genet.

[2] Melchor L, Benítez J (2013). The complex genetic landscape of familial breast cancer. Hum. Genet..

[3] Kessler MD (2016). Challenges and disparities in the application of personalized genomic medicine to populations with African ancestry. Nat. Commun..

[4] Breast Cancer Linkage Consortium. (1997). Pathology of familial breast cancer: differences between breast cancers in carriers of BRCA1 or BRCA2 mutations and sporadic cases. Lancet.

[5] Marotti JD, Schnitt SJ (2018). Genotype-phenotype correlations in breast cancer. Surg. Pathol. Clin..

[6] Riaz N (2017). Pan-cancer analysis of bi-allelic alterations in homologous recombination DNA repair genes. Nat. Commun..

[7] Sun J (2017). Germline mutations in cancer susceptibility genes in a large series of unselected breast cancer patients. Clin. Cancer Res..

[8] Stratton JF (1997). Contribution of BRCA1 mutations to ovarian cancer. N. Engl. J. Med..

[9] Noguchi S (1999). Clinicopathologic analysis of BRCA1- or BRCA2-associated hereditary breast carcinoma in Japanese women. Cancer.

[10] Antoniou A (2003). Average risks of breast and ovarian cancer associated with BRCA1 or BRCA2 mutations detected in case Series unselected for family history: a combined analysis of 22 studies. Am. J. Hum. Genet..

[11] Metcalfe K (2004). Contralateral breast cancer in BRCA1 and BRCA2 mutation carriers. J. Clin. Oncol..

[12] Atchley DP (2008). Clinical and pathologic characteristics of patients with BRCA-positive and BRCA-negative breast cancer. J. Clin. Oncol..

[13] Kaneyasu, T. et al. Datasets and metadata supporting the published article: prevalence of disease-causing genes in Japanese patients with BRCA1/2-wildtype hereditary breast and ovarian cancer syndrome. *figshare*10.6084/m9.figshare.11959233 (2020). 10.1038/s41523-020-0163-1 PMC7293299 32566746

[14] Van der Auwera GA (2013). From FastQ data to high confidence variant calls: the Genome Analysis Toolkit best practices pipeline. Curr. Protoc. Bioinforma..

[15] *ClinVar* https://www.ncbi.nlm.nih.gov/clinvar/submitters/507256/ (2020).

[16] Couch FJ (2017). Associations between cancer predisposition testing panel genes and breast cancer. JAMA Oncol..

[17] Buys SS (2017). A study of over 35,000 women with breast cancer tested with a 25-gene panel of hereditary cancer genes. Cancer.

[18] Lu, H. M. et al. Association of breast and ovarian cancers with predisposition genes identified by large-scale sequencing. *JAMA Oncol*. **5**, 51–57 (2019). 10.1001/jamaoncol.2018.2956 PMC6439764 30128536

[19] Tung N (2016). Frequency of germline mutations in 25 cancer susceptibility genes in a sequential series of patients with breast cancer. J. Clin. Oncol..

[20] Slavin TP (2017). The contribution of pathogenic variants in breast cancer susceptibility genes to familial breast cancer risk. NPJ Breast Cancer.

[21] Girard E (2019). Familial breast cancer and DNA repair genes: Insights into known and novel susceptibility genes from the GENESIS study, and implications for multigene panel testing. Int J. Cancer.

[22] Beitsch PD (2019). Underdiagnosis of hereditary breast cancer: are genetic testing guidelines a tool or an obstacle?. J. Clin. Oncol..

[23] Li J (2016). Targeted massively parallel sequencing of a panel of putative breast cancer susceptibility genes in a large cohort of multiple-case breast and ovarian cancer families. J. Med. Genet..

[24] Castera L (2018). Landscape of pathogenic variations in a panel of 34 genes and cancer risk estimation from 5131 HBOC families. Genet. Med..

[25] Hauke J (2018). Gene panel testing of 5589 BRCA1/2-negative index patients with breast cancer in a routine diagnostic setting: results of the German Consortium for Hereditary Breast and Ovarian Cancer. Cancer Med..

[26] Heikkinen T (2009). The breast cancer susceptibility mutation PALB2 1592delT is associated with an aggressive tumor phenotype. Clin. Cancer Res..

[27] Shimelis H (2018). Triple-negative breast cancer risk genes identified by multigene hereditary cancer panel testing. J. Natl Cancer Inst..

[28] Ohno, S. Identification of responsible genes and development of standardized medicine for familial breast cancer by genetic analysis with NGS technology. *NBDC Human Database*. https://ddbj.nig.ac.jp/jga/viewer/view/study/JGAS00000000224 (2020).

[29] Easton DF (2015). Gene-panel sequencing and the prediction of breast-cancer risk. N. Engl. J. Med..

[30] Nielsen FC, van Overeem Hansen T, Sorensen CS (2016). Hereditary breast and ovarian cancer: new genes in confined pathways. Nat. Rev. Cancer.

[31] Momozawa Y (2018). Germline pathogenic variants of 11 breast cancer genes in 7,051 Japanese patients and 11,241 controls. Nat. Commun..

[32] The CHEK2 Breast Cancer Case-Control Consortium. (2004). CHEK2*1100delC and susceptibility to breast cancer: a collaborative analysis involving 10,860 breast cancer cases and 9,065 controls from 10 studies. Am. J. Hum. Genet..

[33] Kaneko H, Fukao T, Kondo N (2004). The function of RecQ helicase gene family (especially BLM) in DNA recombination and joining. Adv. Biophys..

[34] German J, Sanz MM, Ciocci S, Ye TZ, Ellis NA (2007). Syndrome-causing mutations of the BLM gene in persons in the Bloom’s Syndrome Registry. Hum. Mutat..

[35] Coppa A (2018). Optimizing the identification of risk-relevant mutations by multigene panel testing in selected hereditary breast/ovarian cancer families. Cancer Med..

[36] Ly KI, Blakeley JO (2019). The diagnosis and management of neurofibromatosis type 1. Med. Clin. North Am..

[37] Maani, N. et al. NF1 patients receiving breast cancer screening: insights from The Ontario High Risk Breast Screening Program. *Cancers (Basel)* **11**, 707 (2019). 10.3390/cancers11050707 PMC6562659 31121919

[38] Maxwell KN (2017). BRCA locus-specific loss of heterozygosity in germline BRCA1 and BRCA2 carriers. Nat. Commun..

[39] Tung N (2010). Prevalence and predictors of loss of wild type BRCA1 in estrogen receptor positive and negative BRCA1-associated breast cancers. Breast Cancer Res..

[40] Osorio A (2002). Loss of heterozygosity analysis at theBRCAloci in tumor samples from patients with familial breast cancer. Int. J. Cancer.

[41] Bubien V (2017). Combined tumor genomic profiling and exome sequencing in a breast cancer family implicates ATM in tumorigenesis: a proof of principle study. Genes Chromosomes Cancer.

[42] Renault AL (2018). Morphology and genomic hallmarks of breast tumours developed by ATM deleterious variant carriers. Breast Cancer Res..

[43] Casadei S (2011). Contribution of inherited mutations in the BRCA2-interacting protein PALB2 to familial breast cancer. Cancer Res..

[44] Lee JEA (2018). Molecular analysis of PALB2-associated breast cancers. J. Pathol..

[45] Dworkin AM, Spearman AD, Tseng SY, Sweet K, Toland AE (2009). Methylation not a frequent “second hit” in tumors with germline BRCA mutations. Fam. Cancer.

[46] Nomizu T (1997). Clinicopathological features of hereditary breast cancer. Breast Cancer.

[47] Alemar B (2017). BRCA1 and BRCA2 mutational profile and prevalence in hereditary breast and ovarian cancer (HBOC) probands from Southern Brazil: are international testing criteria appropriate for this specific population?. PLoS ONE.

[48] Arai, M. et al. Genetic and clinical characteristics in Japanese hereditary breast and ovarian cancer: first report after establishment of HBOC registration system in Japan. *J. Hum. Genet.* **63**, 447–457 (2018). 10.1038/s10038-017-0355-1 PMC8716335 29176636

[49] Komoike Y (2005). Analysis of ipsilateral breast tumor recurrences after breast-conserving treatment based on the classification of true recurrences and new primary tumors. Breast Cancer.

[50] Gotoh O (2019). Clinically relevant molecular subtypes and genomic alteration-independent differentiation in gynecologic carcinosarcoma. Nat. Commun..

[51] Li H, Durbin R (2009). Fast and accurate short read alignment with Burrows-Wheeler transform. Bioinformatics.

[52] DePristo MA (2011). A framework for variation discovery and genotyping using next-generation DNA sequencing data. Nat. Genet..

[53] Fromer M (2012). Discovery and statistical genotyping of copy-number variation from whole-exome sequencing depth. Am. J. Hum. Genet..

[54] Gao J (2013). Integrative analysis of complex cancer genomics and clinical profiles using the cBioPortal. Sci. Signal.

[55] Cerami E (2012). The cBio cancer genomics portal: an open platform for exploring multidimensional cancer genomics data. Cancer Discov..

[56] Higasa K (2016). Human genetic variation database, a reference database of genetic variations in the Japanese population. J. Hum. Genet..

[57] Nagasaki M (2015). Rare variant discovery by deep whole-genome sequencing of 1,070 Japanese individuals. Nat. Commun..

[58] Yamaguchi-Kabata Y (2015). iJGVD: an integrative Japanese genome variation database based on whole-genome sequencing. Hum. Genome Var..

[59] Lek M (2016). Analysis of protein-coding genetic variation in 60,706 humans. Nature.

[60] Koboldt DC (2012). VarScan 2: somatic mutation and copy number alteration discovery in cancer by exome sequencing. Genome Res..

[61] Cibulskis K (2013). Sensitive detection of somatic point mutations in impure and heterogeneous cancer samples. Nat. Biotechnol..

[62] McKenna A (2010). The Genome Analysis Toolkit: a MapReduce framework for analyzing next-generation DNA sequencing data. Genome Res..

[63] Magi A (2013). EXCAVATOR: detecting copy number variants from whole-exome sequencing data. Genome Biol..

[64] Patch AM (2015). Whole-genome characterization of chemoresistant ovarian cancer. Nature.

[65] Richards S (2015). Standards and guidelines for the interpretation of sequence variants: a joint consensus recommendation of the American College of Medical Genetics and Genomics and the Association for Molecular Pathology. Genet. Med..

[66] Maxwell KN (2016). Evaluation of ACMG-guideline-based variant classification of cancer susceptibility and non-cancer-associated genes in families affected by breast cancer. Am. J. Hum. Genet..

