# Supplementary material for: Prevalence of disease-causing genes in Japanese patients with BRCA1/2-wildtype hereditary breast and ovarian cancer syndrome
Source: NPJ Breast Cancer. 2020 Jun 12;6:25. doi: 10.1038/s41523-020-0163-1 (PMC7293299; doi:10.1038/s41523-020-0163-1)
Supplement: Supplementary file 1 — Supplementary [file 41523_2020_163_MOESM1_ESM.pdf]

Prevalence of Disease-Causing Genes in Japanese Patients with *BRCA1/2*-Wildtype Hereditary Breast and Ovarian Cancer Syndrome

Tomoko Kaneyasu[1#], Seiichi Mori[1#], Hideko Yamauchi[2], Shozo Ohsumi[3], Shinji Ohno[4], Daisuke Aoki[5], Shinichi Baba[6], Junko Kawano[6], Yoshio Miki[1], Naomichi Matsumoto[7], Masao Nagasaki[8], Reiko Yoshida[9], Sadako Akashi-Tanaka[10], Takuji Iwase[4], Dai Kitagawa[4], Kenta Masuda[5], Akira Hirasawa[5], Masami Arai[9], Junko Takei[2], Yoshimi Ide[10], Osamu Gotoh[1], Noriko Yaguchi[1], Mitsuyo Nishi[6], Keika Kaneko[3], Yumi Matsuyama[3], Megumi Okawa[2], Misato Suzuki[2], Aya Nezu[4], Shiro Yokoyama[10], Sayuri Amino[1], Mayuko Inuzuka[10], Tetsuo Noda[11] and Seigo Nakamura[10]

1. Project for Development of Innovative Research on Cancer Therapeutics, Cancer Precision Medicine Center, Japanese Foundation for Cancer Research, 3-8-31 Ariake, Koto-ku, Tokyo, Japan.
2. Department of Breast Surgical Oncology, St. Luke’s International Hospital, 10-1 Akashi-cho, Chuo-ku, Tokyo, Japan.
3. National Hospital Organization Shikoku Cancer Center, 160 Kou, Minamiumemoto-machi, Matsuyama, Ehime, Japan.

4. Breast Oncology Center, Cancer Institute Hospital, Japanese Foundation for Cancer Research, 3-8-31 Ariake, Koto-ku, Tokyo, Japan.

5. Department of Obstetrics & Gynecology, Keio University School of Medicine, 35 Shinano-cho, Shinjuku-ku, Tokyo, Japan.

6. Sagara Hospital, 3-31 Matsubara-cho, Kagoshima, Japan.

7. Department of Human Genetics, Yokohama City University Graduate School of Medicine, Fukuura 3-9, Kanazawa-ku, Yokohama, Japan.

8. Department of Integrative Genomics, Tohoku Medical Megabank Organization, Tohoku University, 2-1, Seiryo-machi, Aoba-ku, Sendai, Miyagi, Japan.

9. Department of Clinical Genetic Oncology, Cancer Institute Hospital, Japanese Foundation for Cancer Research, 3-8-31 Ariake, Koto-ku, Tokyo, Japan.

10. Division of Breast Surgical Oncology, Showa University School of Medicine, 1-5-8 Hatanodai, Shinagawa-ku, Tokyo, Japan.

11. Cancer Institute, Japanese Foundation for Cancer Research, 3-8-31 Ariake, Koto-ku, Tokyo, Japan.

[#]These authors contributed equally to this work.

Supplementary Methods

Gene selection for target panel analysis

Based on the significance of causality in hereditary breast and ovarian cancer (HBOC) syndrome, we focused on a combination of 119 genes selected from the following three groups (Supplementary Table 1): 1) Well-recognized, disease-causing genes, described as actionable genes in the NCCN guidelines, ver. 3.2019, for Genetic/Familial High-Risk Assessment: Breast and Ovarian (tier 1, 20 genes): *BRCA1*, *BRCA2*, *STK11*, *PTEN*, *CDH1*, *NF1*, *TP53*, *NBN*, *ATM*, *CHEK2*, *PALB2*, *BRIP1*, *BARD1*, *RAD51C*, *RAD51D*, *EPCAM*, *MLH1*, *MSH2*, *MSH6* and *PMS2*;[1,2] 2) Genes reported as disease-causing (tier 2, 10 genes): *RAD50*, *XRCC2*, *RAD51B*, *MRE11A*, *FANCC*, *BLM*, *FAM175A*, *RINT1*, *FANCM* and *RECQL*;[3,4] 3) Genes that constitute the homologous recombination (HR) pathway[5,6] and driver genes on miscellaneous academic and commercial NGS panels relevant to breast, ovarian, and other cancer types[7,8] (tier 3, 89 genes; Supplementary Table 1). A custom panel of these 119 genes was designed with SureDesign (Agilent Technologies). Among the 119 genes, variants on 28 genes other than *BRCA1* and *BRCA2* of the tier-1 and -2 categories were subjected to pathogenicity classification, as described below.

Supplementary Note 1

Validity of the 2-level criteria for inclusion

The 2-level (HBOC history level) criteria for eligibility in the current study was validated by comparing against the National Comprehensive Cancer Network (NCCN) genetic testing criteria[2] (Supplementary Figures 1A and 1B). If the patient met the criteria, the number of categories was counted and tallied. Among the 24 categories, we did not use 11 categories that were based on or around the following data: patient or family member Gleason score; presence or absence of prostate cancer metastasis (the database we used for clinical information did not contain the detailed prostate cancer information); Ashkenazi Jew diaspora (all study subjects were Japanese); tumor testing (all subjects did not receive any panel test for their tumors); and a lack of family history (all subjects had family history). All individual patients in this study met 2 to 6 of the 13 categories. *BRCA1*-mutant patients had significantly more categories than *BRCA2*-mutant and *BRCA1/2*-wildtype cases (Fisher exact tests; $p < 0.01$ and $p < 0.01$, respectively). More *BRCA1*/*2*-positive patients were allocated as HBOC history level 1 than level 2, consistent with the strength of the family history (Fisher exact test; $p = 0.01$, Figure 2A).

Supplementary Note 2

Development of pathogenicity classification pipeline

As per a previous method and based on the guidelines developed by the American College of Medical Genetics and Genomics (ACMG) and the Association for Molecular Pathology (AMP),[9,10] we constructed a 5-category pathogenicity classification pipeline to determine “true” deleterious variants from variable SNVs and indels detected among 28 disease-causing genes (Supplementary Table 1, Supplementary Figure 2 and Methods). To assess the reliability of the methodology, we applied the pipeline to 39 and 68 variants in *BRCA1* and *BRCA2* alleles, respectively, identified from the exome-sequenced data of 14 *BRCA1*-mutated, 5 *BRCA1* variant of uncertain significance (VUS), 13 *BRCA2*-mutated, 5 *BRCA2* VUS, and 67 *BRCA1/2*-wildtype patients (Figure 1). After manual adjustment, the resultant calls by the pipeline were then compared with those called by commercial companies. In the assessments with the 5-categorical system (P, LP, VUS, LB and B) between the “ACMG-AMP calls” and “commercial company calls”, only 9 (23.1%) of 39 *BRCA1* variants and 7 (10.4%) of 67 *BRCA2* variants matched. However, the concordance improved to 24 (61.5%) of 39 *BRCA1* variants and 48 (71.6%) of 67 *BRCA2* variants when we used the 3-level classification (P/LP, VUS and LB/B, see Methods). Of particular interest, there was a 100% match for all of the 14 *BRCA1*

and 13 *BRCA2* P/LPs between the ACMG-AMP and commercial company calls. Whereas 5 *BRCA1* and 31 *BRCA2* LB/B ACMG-AMP calls were LB/B in commercial company calls, 15 *BRCA1* and 19 *BRCA2* LB/B commercial company calls were assigned as VUS in the ACMG-AMP calls. From these assessments, we concluded that our pathogenicity classification system following the ACMG-AMP guidelines could produce sufficiently reliable calls for P/LP variants but not for LB/B assignments.

Supplementary Note 3

Patient and tumor features associated with P/LP other gene (non-*BRCA1/2*) variants

To identify factors that could possibly distinguish between patients with a non-*BRCA1/2* pathogenic variant and those without such a mutation, we performed logistic regression analyses for pathogenic variants against multiple clinicopathological parameters (age at onset, first tumor histology, first tumor molecular subtype, first tumor nuclear grade, HBOC history level, laterality, and type of additional cancer). The analyses revealed a significant correlation between pathogenic variant and age at onset: a younger onset was associated with these other gene (non-*BRCA1/2*) mutations (univariate model: p = 0.02 and z-value = -2.3; multivariate model: p = 0.03 and z-value = -2.2). However, the accuracy in the predictive model based on this association was low (AUC [area under the curve] = 0.61 by the receiver operating characteristic analysis), which implies that the age at onset cannot be used as a predictor.

Supplementary Note 4

Family member analysis

Thirty-four family member germline samples were available for 13 index cases, and these samples were subjected to targeted re-sequencing analysis with the 119-gene panel (Supplementary Figure 6). Pedigree charts were drawn with family member history for cancer and mutational status for 28 HBOC syndrome disease-causing genes. In 4 families, the mutant and wildtype alleles showed exact concordance with breast cancer occurrence (*PALB2* p.P1086* of A0139, *BLM* p.G512fs of B0187, *ATM* c.4776+2T>A of B0242 and *BRIP1* p.A1081fs of B0285 in Supplementary Figure 6). The remaining 9 families did not perfectly match with the presence of breast or ovarian disease. Specifically, in the A0277 family, a *CHEK2* p.L486fs mutation was detected not only for the proband but also for her younger sister, who had not exhibited cancer by the age of 33 when she was tested. B0273, E0237, and B0288 families showed a similar pattern: mutant carrier females had not developed breast or ovarian cancer by the age at testing, which may be due to the low penetrance of these variants. In the C0120 family, a *RAD51D* deleterious mutation (*RAD51D* p.K111fs) found in the proband was associated with her father (who had gastric cancer) but not with her mother (who had breast cancer). The *BARD1* exon 5–7 deletion in the E0114 family was associated with the proband but not with her

mother or her daughter, both of whom presented with a history of breast cancer. This type of discordance was also observed in the D0231 and B0288 families, possibly because of the coincident occurrence of sporadic breast cancer in the families. In the A0281 family, the proband and her older sister had different mutant alleles in *BRIP1* p.R356* and *MRE11A* p.N511fs in an exclusive manner.

The small sizes of the families (median: 3, range: 2–6 family members, including the proband) and the small number of affected and unaffected individuals hampered performing an accurate linkage analysis. Based on the assumption that HBOC syndrome is inherited in an autosomal dominant mode, paramlink (ver. 1.1.2)[11] was used to run MERLIN[12] for computation of the LOD (logarithm of the odds) score per variant per family. Penetrance and recombination rates were simultaneously estimated by maximizing LOD score[13] using the constrOptim function on R (ver. 3.3.1). The LOD scores for the 13 families ranged from 0 to 0.6 (median: 0), which were far below the significance threshold of 3.

These family member analyses provided an imperfect match between the mutational status of genes and the affected or unaffected family members; a mutant gene was not necessarily shared by a family member with breast or ovarian cancer, and an unaffected member or a member with non-breast and non-ovarian cancer often retained the same mutation as the index patient. Such complexity has been consistently observed[14,15] and is perhaps derived from the low penetrance of a gene with multiple

individual gene involvement in HBOC syndrome. These discrepancies could critically impact any future preventive medical care for family members. For example, in our cohort, affected sisters had different genes with mutations (*BRIP1* and *MRE11A*). This implies a need to test a panel of genes and not just a single site on the gene of interest. Additionally, because the risk of developing breast, ovarian or other cancer is largely unknown for many variants of genes other than *BRCA1/2*, there is no definitive preventive medical care program for carriers, including males. As such, there is a need to follow up these carriers for the detection of future cancers. Family member analyses in the current study are thus useful for providing a biological basis for future clinical intervention.

Supplementary Figures

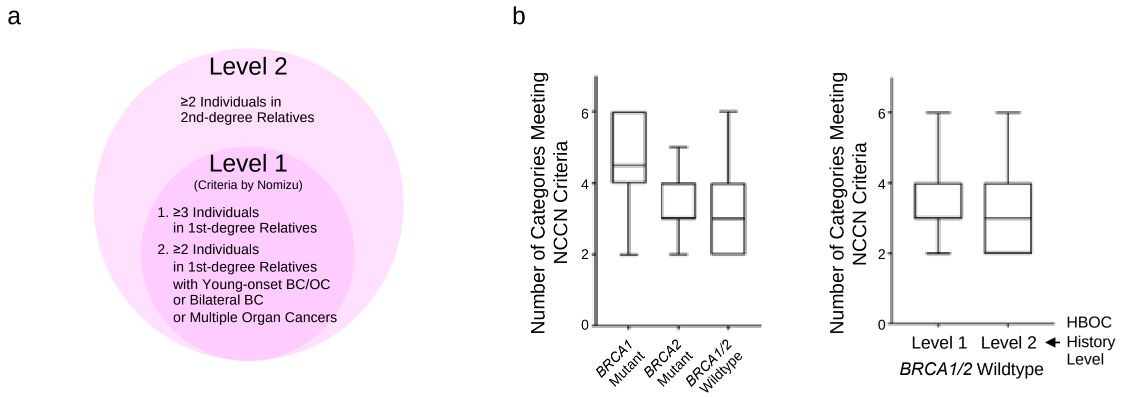

Supplementary Figure 1. Diagnostic criteria and *BRCA1/2* positivity.

a. Two-level diagnostic criteria used in this study of hereditary breast and ovarian cancer (HBOC) (history levels 1 and 2). See also the definitions described in the Methods. **b**. Comparison of the 2-level criteria with the National Comprehensive Cancer Network (NCCN) genetic test criteria. Graphs show the number of NCCN criteria for *BRCA1/2* mutational status versus wildtype (left) and the number of NCCN criteria at the HBOC history levels (1 and 2; right). For each patient that met the criteria, the number of NCCN criteria was counted. Numbers were tallied for each group. Data are presented as box and whisker plots, with the box indicating the 25th and 75th percentiles, the middle bar indicating the median, and the whiskers indicating the minimal and maximal numbers.

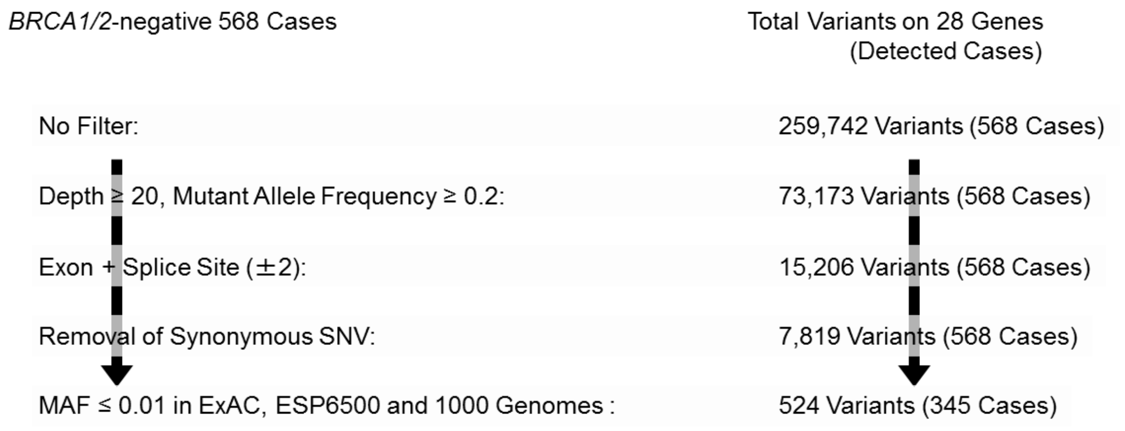

Supplementary Figure 2. Filters and number of variants used in the analysis of 28 genes among 568 *BRCA1/2* mutation-negative (wildtype) cases. The number of cases with the detected variants is shown in parentheses.

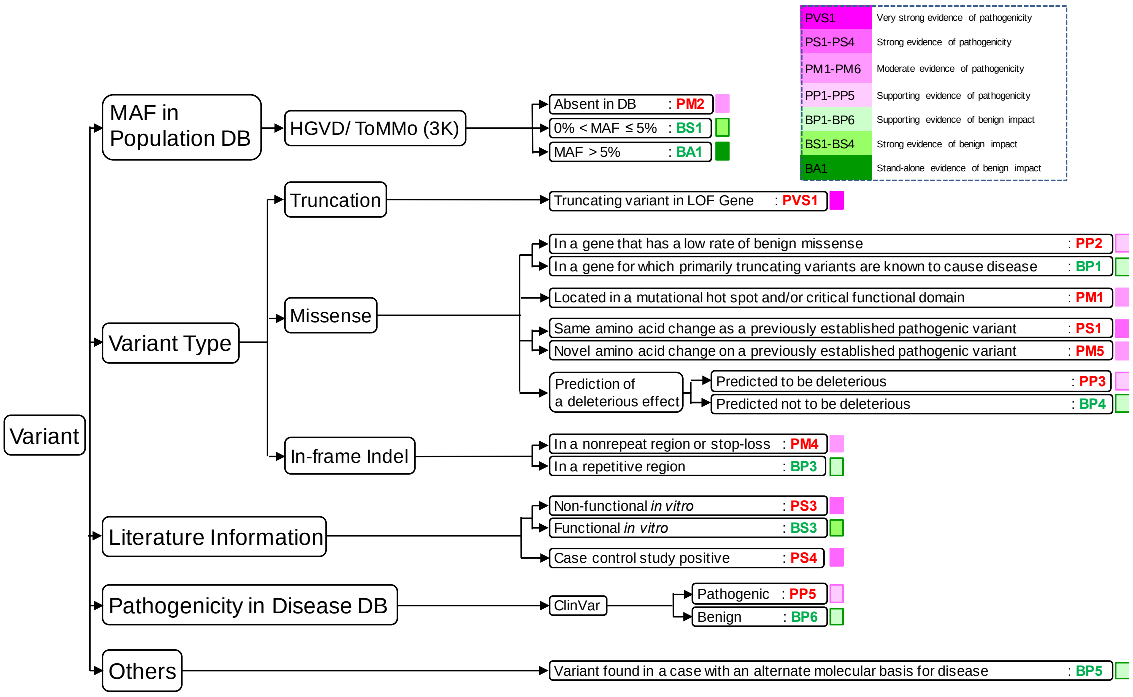

Supplementary Figure 3. Pathogenicity classification algorithm following the American College of Medical Genetics and Genomics–Association for Molecular Pathology (ACMG-AMP) guidelines.

Case	Breast /Ovary	Gene	Nucleotide Substitution Large Deletion	Amino Acid Substitution
A1013	Breast	*PALB2*	c.2167_2168del	p.M723fs
A0139	Breast	*PALB2*	c.C3256T	p.R1086*
B0215	Breast	*PALB2*	c.C3256T	p.R1086*
A0214	Breast	*PALB2*	c.G1384T	p.E462*
B0219	Breast	*PALB2*	c.G1384T	p.E462*
B0277	Breast	*PALB2*	c.T1451G	p.L484*
A0338	Breast	*PALB2*	c.820dupA	p.T274fs
D0241	Ovary	*ATM*	c.C2413T	p.R805*
E0125	Breast	*ATM*	c.240dupA	p.P80fs
A0346	Breast	*ATM*	c.1121_1122del	p.Q374fs
B0242	Breast	*ATM*	c.4776+2T>A	Splicing
C0158	Breast	*ATM*	c.5509_5510del	p.F1837fs
A0301	Breast	*BARD1*	c.C1921T	p.R641*
A0331	Breast	*BARD1*	c.C448T	p.R150*
		ATM	c.7878_7882del	p.A2626fs
A0298	Breast	*BARD1*	c.C1345T	p.Q449*
B0258	Breast	*BARD1*	c.518dupC	p.A173fs
E0114	Breast	*BARD1*	Exon 5-7 Deletion	
C0120	Breast	*RAD51D*	c.331_332insTA	p.K111fs
D0239	Ovary	*RAD51D*	c.331_332insTA	p.K111fs
A0231	Breast	*RAD51D*	c.454delG	p.V152fs
B0220	Breast	*RAD51D*	c.C445T	p.Q149*
D0221	Breast	*BLM*	c.319dupT	p.S106fs
C0256	Breast	*BLM*	c.319dupT	p.S106fs
B0187	Breast	*BLM*	c.1536dupA	p.G512fs
B0292	Breast	*BLM*	c.3751+2T>C	Splicing
A0281	Breast	*BRIP1*	c.C1066T	p.R356*
B0285	Breast	*BRIP1*	c.3240dupT	p.A1081fs
B0170	Breast	*BRIP1*	c.918+2T>C	Splicing
E0237	Breast	*RAD51C*	c.G133T	p.E45*
C0206	Breast	*RAD51C*	Exon 6-9 Deletion	
B0263	Breast	*RAD51C*	Exon 6-9 Deletion	
B0288	Breast	*FANCM*	c.2190_2191insCT	p.Q730fs
D0231	Breast	*FANCM*	c.2521_2524del	p.K841fs
E0131	Breast	*RAD50*	c.1633dupA	p.D544fs
B0198	Breast	*NF1*	c.765delT	p.G255fs
A0277	Breast	*CHEK2*	c.1455dupG	p.L486fs
D0222	Breast	*RECQL*	c.1548dupT	p.D517_S518delins*

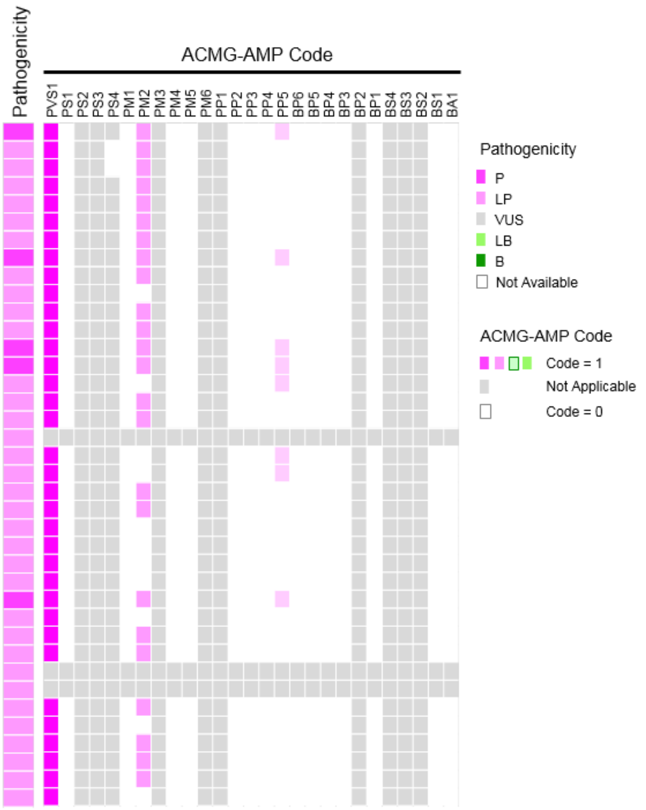

Supplementary Figure 4. Thirty-eight pathogenic or likely pathogenic variants identified in the previously known 28 disease-causing genes (excluding *BRCA1/2*) for HBOC in 37 of 568 patients. The variants included: 19 frameshift indels, 3 CNVs, 13 stopgain SNV/indels, and 3 splice site SNVs. The results from the pathogenicity classification according to the ACMG-AMP guidelines is shown. Presence (code = 1; graded green or pink) or absence (code = 0; white) of classification codes is shown. Grey was used when the code information was not available. Pathogenicity classifications are shown as pink (P: pathogenic), light pink (LP: likely pathogenic), grey (VUS: variant of uncertain significance), light green (LB: likely benign) and green (B: benign). Since the ACMG-AMP guidelines do not cover CNVs, 3 copy number loss variants have neither ACMG-AMP codes nor ACMG-AMP raw calls. “Pathogenicity” indicates the final call after manual inspection. ACMG-AMP, American College of Medical Genetics and Genomics and the Association for Molecular Pathology; HBOC, hereditary breast and ovarian cancer syndrome.

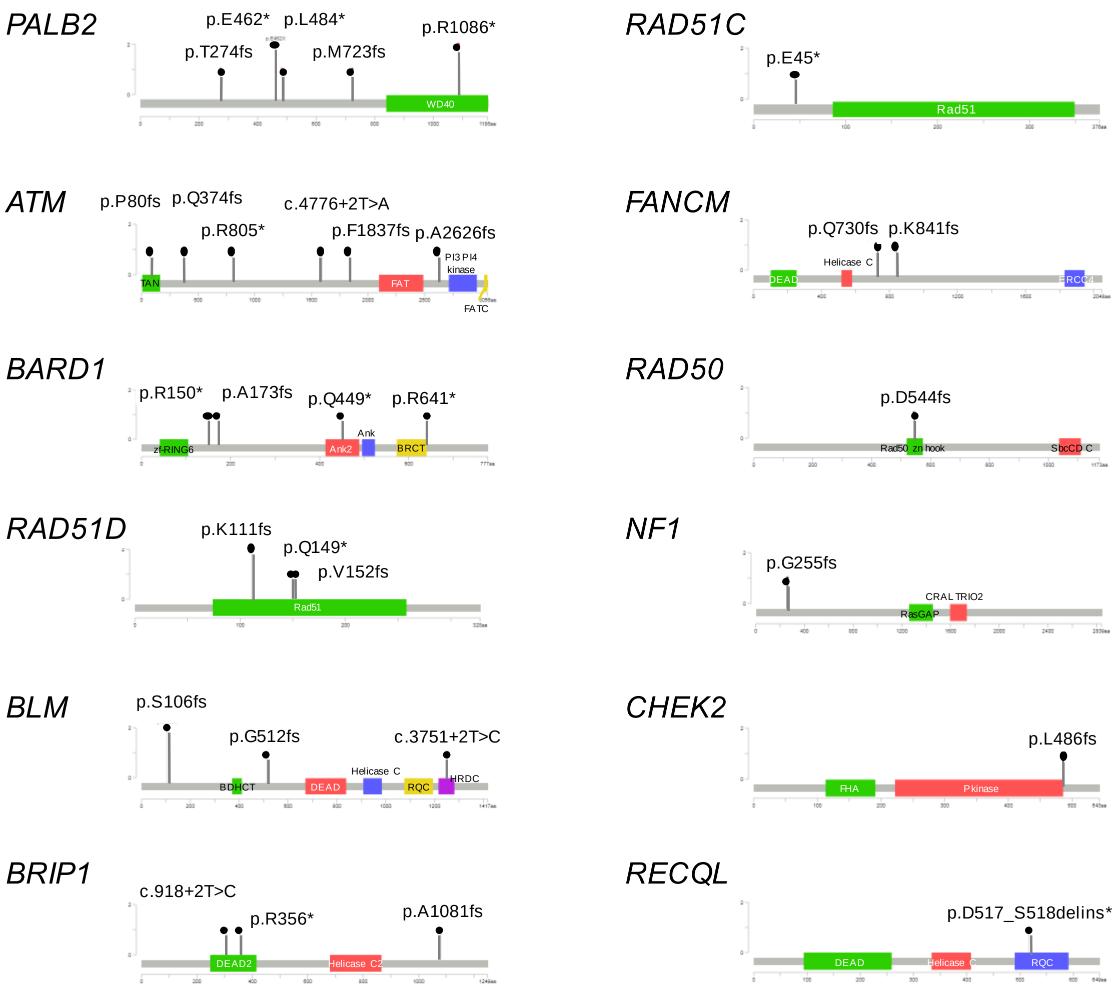

Supplementary Figure 5. Locations of the variants in each gene in the lollipop mutation diagram. Locations of 19 frameshift indels, 13 stopgain SNV/indels and 3 splice site SNVs in the gene with Pfam domain[16] are shown.

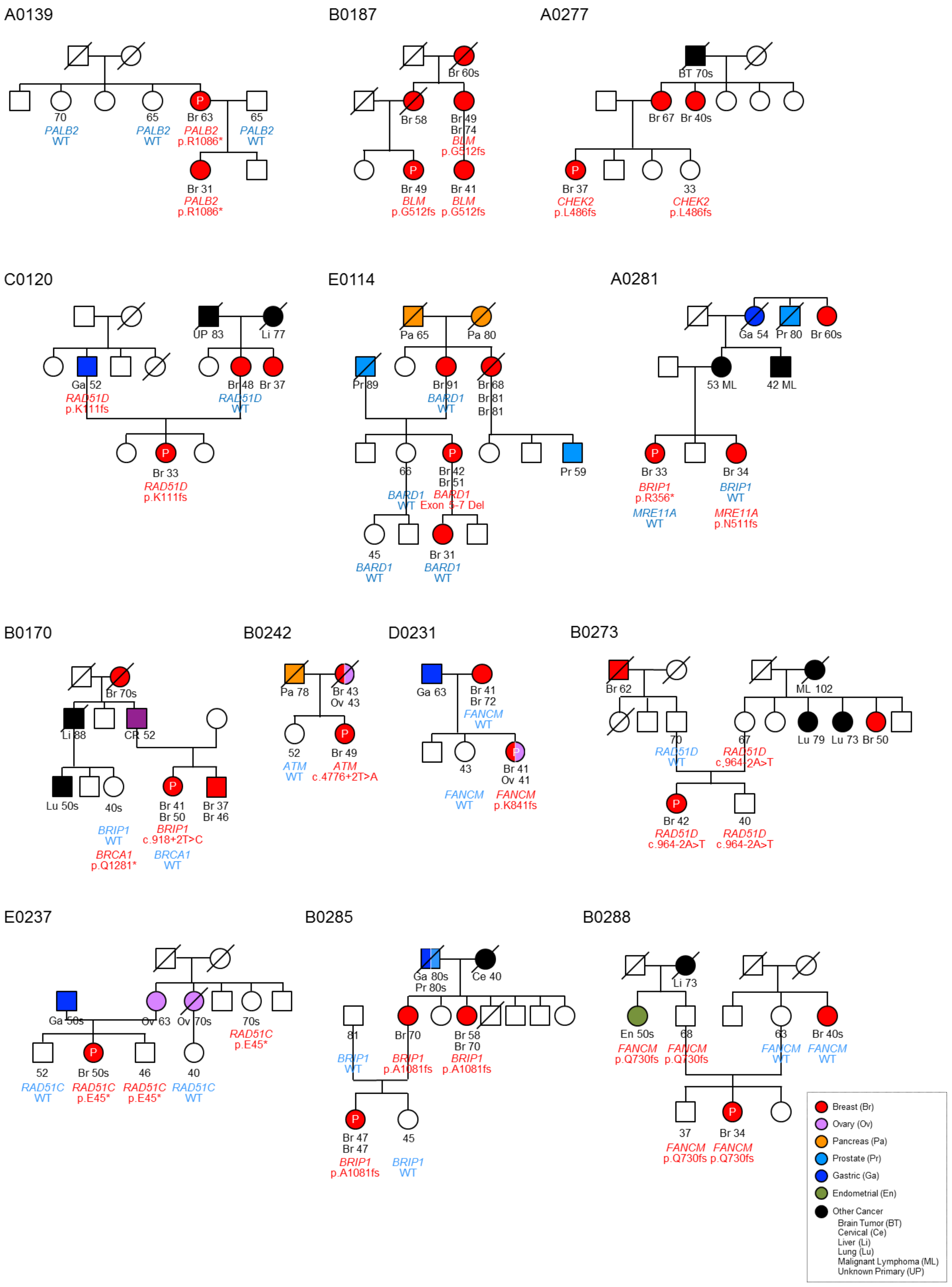

A0139
70
PALB2
WT
65
PALB2
WT
P
Br 63
PALB2
p.R1086*
65
PALB2
WT
P
Br 31
PALB2
p.R1086*
B0187
Br 60s
Br 58
Br 49
Br 74
BLM
p.G512fs
P
Br 49
BLM
p.G512fs
Br 41
BLM
p.G512fs
A0277
BT 70s
Br 67
Br 40s
P
Br 37
CHEK2
p.L486fs
33
CHEK2
p.L486fs
C0120
UP 83
Li 77
Ga 52
RAD51D
p.K111fs
Br 48
Br 37
RAD51D
WT
P
Br 33
RAD51D
p.K111fs
E0114
Pa 65
Pa 80
Pr 89
Br 91
BARD1
WT
Br 68
Br 81
Br 81
66
BARD1
WT
P
Br 42
Br 51
BARD1
Exon 5-7 Del
Pr 59
45
BARD1
WT
Br 31
BARD1
WT
A0281
Ga 54
Pr 80
Br 60s
53 ML
42 ML
P
Br 33
BRIP1
p.R356*
MRE11A
WT
Br 34
BRIP1
WT
MRE11A
p.N511fs
B0170
Br 70s
Li 88
CR 52
Lu 50s
40s
BRIP1
WT
BRCA1
p.Q1281*
P
Br 41
Br 50
BRIP1
c.918+2T>C
BRCA1
WT
Br 37
Br 46
B0242
Pa 78
Br 43
Ov 43
52
ATM
WT
P
Br 49
ATM
c.4776+2T>A
D0231
Ga 63
Br 41
Br 72
FANCM
WT
43
FANCM
WT
P
Br 41
Ov 41
FANCM
p.K841fs
B0273
Br 62
ML 102
70
RAD51D
WT
67
RAD51D
c.964-2A>T
Lu 79
Lu 73
Br 50
P
Br 42
RAD51D
c.964-2A>T
40
RAD51D
c.964-2A>T
E0237
Ga 50s
Ov 63
Ov 70s
70s
RAD51C
p.E45*
52
RAD51C
WT
P
Br 50s
RAD51C
p.E45*
46
RAD51C
p.E45*
40
RAD51C
WT
B0285
Ga 80s
Pr 80s
Ce 40
81
BRIP1
WT
Br 70
BRIP1
p.A1081fs
Br 58
Br 70
BRIP1
p.A1081fs
P
Br 47
Br 47
BRIP1
p.A1081fs
45
BRIP1
WT
B0288
Li 73
En 50s
FANCM
p.Q730fs
68
FANCM
p.Q730fs
63
FANCM
WT
Br 40s
FANCM
WT
37
FANCM
p.Q730fs
P
Br 34
FANCM
p.Q730fs
Breast (Br)
Ovary (Ov)
Pancreas (Pa)
Prostate (Pr)
Gastric (Ga)
Endometrial (En)
Other Cancer
Brain Tumor (BT)
Cervical (Ce)
Liver (Li)
Lung (Lu)
Malignant Lymphoma (ML)
Unknown Primary (UP)

Supplementary Figure 6. Pedigree charts of patients with pathogenic or likely pathogenic variants, who also have family member variant information. Females and males are represented as circles and squares, respectively. A diagonal line through the shape indicates a deceased person at the time the pedigree chart was drawn. Red, purple, orange, sky-blue, blue, olive, and black are used to indicate breast, ovarian, pancreas, prostate, gastric, endometrial and the other tissue cancers. The number at the bottom indicates the age of onset of cancer if the family member had cancer history, or age when the member received a genetic test for the variant. Genetic information is shown beneath each family member when the member received a genetic test for the variant. Red and sky-blue fonts are used to indicate the mutant and wildtype alleles for the variant, respectively.

Supplementary Table 1. List of 119 HBOC-related genes*

No.	Gene	NCCN Guidelines: Breast[2]	NCCN Guidelines: Ovary[2]	Easton 2015[1]	Nielsen 2016[4]	HR Pathway[5,6]	Miscellaneous Academia and Commercial Panels[17-25]	Tier
1	*BRCA1*	YES	YES	YES	YES	YES	YES	1
2	*BRCA2*	YES	YES	YES	YES	YES	YES	1
3	*STK11*	YES	YES	YES			YES	1
4	*NBN*	YES		YES	YES	YES	YES	1
5	*ATM*	YES		YES	YES		YES	1
6	*CHEK2*	YES		YES	YES		YES	1
7	*PALB2*	YES		YES	YES		YES	1
8	*TP53*	YES		YES	YES		YES	1
9	*PTEN*	YES		YES	YES		YES	1
10	*BARD1*	YES		YES	YES		YES	1
11	*CDH1*	YES		YES			YES	1
12	*NF1*	YES		YES			YES	1
13	*RAD51D*		YES		YES	YES	YES	1
14	*RAD51C*		YES		YES		YES	1
15	*BRIP1*		YES		YES		YES	1
16	*EPCAM*		YES				YES	1
17	*MLH1*		YES				YES	1
18	*MSH2*		YES				YES	1
19	*MSH6*		YES				YES	1
20	*PMS2*		YES				YES	1
21	*RAD50*				YES	YES	YES	2
22	*XRCC2*				YES	YES	YES	2
23	*RAD51B*				YES	YES	YES	2
24	*MRE11A*				YES	YES	YES	2
25	*FANCC*				YES	YES	YES	2
26	*BLM*				YES	YES	YES	2
27	*FAM175A*				YES		YES	2
28	*RINT1*				YES		YES	2
29	*FANCM*				YES		YES	2
30	*RECQL*				YES		YES	2

No.	Gene	NCCN Guidelines: Breast[2]	NCCN Guidelines: Ovary[2]	Easton 2015[1]	Nielsen 2016[4]	HR Pathway[5,6]	Miscellaneous Academia and Commercial Panels[17-25]	Tier
31	*RAD51*					YES	YES	3
32	*XRCC3*					YES	YES	3
33	*FANCA*					YES	YES	3
34	*FANCI*					YES	YES	3
35	*FANCL*					YES	YES	3
36	*RBBP8*					YES	YES	3
37	*CDKN1A*					YES		3
38	*ATR*						YES	3
39	*TP53BP1*						YES	3
40	*FANCB*					YES	YES	3
41	*FANCD2*					YES	YES	3
42	*FANCE*					YES	YES	3
43	*FANCF*					YES	YES	3
44	*FANCG*					YES	YES	3
45	*GEN1*					YES	YES	3
46	*SHFM1*					YES	YES	3
47	*RAD52*					YES		3
48	*EME1*					YES		3
49	*EME2*					YES		3
50	*MUS81*					YES		3
51	*C19orf40*					YES		3
52	*MSH4*					YES		3
53	*MSH5*					YES		3
54	*RAD54B*					YES		3
55	*RAD54L*					YES		3
56	*DMC1*					YES		3
57	*SLX1A*					YES		3
58	*SLX1B*					YES		3
59	*CCNA1*					YES		3
60	*CCNA2*					YES		3

No.	Gene	NCCN Guidelines: Breast[2]	NCCN Guidelines: Ovary[2]	Easton 2015[1]	Nielsen 2016[4]	HR Pathway[5,6]	Miscellaneous Academia and Commercial Panels[17-25]	Tier
61	*CCNB1*					YES		3
62	*CCNB2*					YES		3
63	*CCNB3*					YES		3
64	*CDC25C*					YES		3
65	*CDK1*					YES		3
66	*CDK2*					YES		3
67	*CDK6*					YES		3
68	*CSNK2A1*					YES		3
69	*CSNK2A2*					YES		3
70	*CSNK2A3*					YES		3
71	*CSNK2B*					YES		3
72	*MDC1*					YES		3
73	*MORF4L1*					YES		3
74	*PLK1*					YES		3
75	*MUTYH*						YES	3
76	*SMARCA4*						YES	3
77	*CDK4*						YES	3
78	*APC*						YES	3
79	*BMPR1A*						YES	3
80	*CDKN2A*						YES	3
81	*POLD1*						YES	3
82	*SMAD4*						YES	3
83	*GREM1*						YES	3
84	*POLE*						YES	3
85	*AKT1*						YES	3
86	*AXIN2*						YES	3
87	*BAP1*						YES	3
88	*CDC73*						YES	3
89	*DICER1*						YES	3
90	*HOXB13*						YES	3

No.	Gene	NCCN Guidelines: Breast[2]	NCCN Guidelines: Ovary[2]	Easton 2015[1]	Nielsen 2016[4]	HR Pathway[5,6]	Miscellaneous Academia and Commercial Panels[17-25]	Tier
91	*MEN1*						YES	3
92	*PIK3CA*						YES	3
93	*PMS1*						YES	3
94	*SDHB*						YES	3
95	*SDHD*						YES	3
96	*VHL*						YES	3
97	*CTNNB1*						YES	3
98	*H2AFX*						YES	3
99	*RPA1*						YES	3
100	*RPA2*						YES	3
101	*RPA3*						YES	3
102	*RPA4*						YES	3
103	*SLX4*						YES	3
104	*BUB1B*						YES	3
105	*KIT*						YES	3
106	*LIG4*						YES	3
107	*MET*						YES	3
108	*MLH3*						YES	3
109	*PALLD*						YES	3
110	*PPM1D*						YES	3
111	*PRSS1*						YES	3
112	*PTCH1*						YES	3
113	*RECQL4*						YES	3
114	*RECQL5*						YES	3
115	*RET*						YES	3
116	*RIF1*						YES	3
117	*SPINK1*						YES	3
118	*UIMC1*						YES	3
119	*WRN*						YES	3

*HBOC-related genes refers to well-recognized HBOC-causing genes, genes reported as HBOC-causing, and other relevant cancer genes.

Supplementary References

1 Easton, D. F. *et al.* Gene-panel sequencing and the prediction of breast-cancer risk. *N Engl J Med* **372**, 2243-2257 (2015).

2 National Comprehensive Cancer Network. NCCN clinical practice guidelines in oncology, genetic/familial high-risk assessment: Breast and ovarian (version 3 2019).

3 Melchor, L. & Benítez, J. The complex genetic landscape of familial breast cancer. *Human Genetics* **132**, 845-863 (2013).

4 Nielsen, F. C., van Overeem Hansen, T. & Sorensen, C. S. Hereditary breast and ovarian cancer: new genes in confined pathways. *Nat Rev Cancer* **16**, 599-612 (2016).

5 Milanowska, K. *et al.* REPAIRtoire--a database of DNA repair pathways. *Nucleic Acids Res* **39**, D788-792 (2011).

6 Wood, R. D., Mitchell, M. & Lindahl, T. Human DNA repair genes, 2005. *Mutat Res* **577**, 275-283 (2005).

7 Zehir, A. *et al.* Mutational landscape of metastatic cancer revealed from prospective clinical sequencing of 10,000 patients. *Nat Med* **23**, 703-713 (2017).

8 Meric-Bernstam, F. *et al.* Feasibility of Large-Scale Genomic Testing to Facilitate Enrollment

Onto Genomically Matched Clinical Trials. *J Clin Oncol* **33**, 2753-2762 (2015).

9 Maxwell, K. N. *et al.* Evaluation of ACMG-Guideline-Based Variant Classification of Cancer Susceptibility and Non-Cancer-Associated Genes in Families Affected by Breast Cancer. *Am J Hum Genet* **98**, 801-817 (2016).

10 Richards, S. *et al.* Standards and guidelines for the interpretation of sequence variants: a joint consensus recommendation of the American College of Medical Genetics and Genomics and the Association for Molecular Pathology. *Genet Med* **17**, 405-424 (2015).

11 https://CRAN.R-project.org/package=paramlink.

12 Abecasis, G. R., Cherny, S. S., Cookson, W. O. & Cardon, L. R. Merlin--rapid analysis of dense genetic maps using sparse gene flow trees. *Nat Genet* **30**, 97-101 (2002).

13 Hodge, S. E. & Elston, R. C. Lods, wrods, and mods: the interpretation of lod scores calculated under different models. *Genet Epidemiol* **11**, 329-342 (1994).

14 Li, J. *et al.* Targeted massively parallel sequencing of a panel of putative breast cancer susceptibility genes in a large cohort of multiple-case breast and ovarian cancer families. *J Med Genet* **53**, 34-42 (2016).

15 Coppa, A. *et al.* Optimizing the identification of risk-relevant mutations by multigene panel

testing in selected hereditary breast/ovarian cancer families. *Cancer Med* **7**, 46-55 (2018).

16 https://pfam.xfam.org/.

17 http://www.ambrygen.com/tests/ovanext.

18 https://www.breasthealthuk.com/screeningservices/genetictesting/breastgene.

19 https://www.centoportal.com/order/new/test-catalog/analysis-method.

20 http://geneticslab.emory.edu/tests/MM208.

21 https://fulgentdiagnostics.com/test/Breast-Ovarian-Cancer.

22 http://www.genedx.com/test-catalog/available-tests/breastovarian-cancer-panel/.

23 https://www.invitae.com/en/physician/tests/01201/?cat=CAT000015.

24 https://www.myriadpro.com/myrisk/why-myriad-myrisk/gene-selection/.

25 http://web.labmed.washington.edu/tests/genetics/BROCA.
